# The 22nd Annual Meeting of the Rocky Mountain Virology Association

**DOI:** 10.3390/v15010098

**Published:** 2022-12-29

**Authors:** Oshani C. Ratnayake, Paul Gendler, Benjamin Swartzwelter, Alexandra Keene, Ali L. Brehm, Sandra L. Quackenbush, Joel Rovnak, Rushika Perera

**Affiliations:** 1 Center for Vector-Borne Infectious Diseases, Department of Microbiology, Immunology, and Pathology, Colorado State University, Fort Collins, CO 80524, USA; 2 Center for Metabolism of Infectious Diseases (C4MInD), Colorado State University, Fort Collins, CO 80523, USA; 3 Department of Molecular, Cellular, and Development Biology, University of Colorado Boulder, Boulder, CO 80302, USA; 4 Department of Microbiology, Immunology, and Pathology, Colorado State University, Fort Collins, CO 80523, USA

**Keywords:** flavivirus, SARS-CoV-2, host–virus interactions, prions, extracellular vesicles, immune response, science communication, coronaviruses

## Abstract

Following the cause established twenty-two years ago, the 22nd Annual Rocky Mountain Virology Association meeting was held amidst the resplendent Rocky Mountains within the Arapahoe and Roosevelt National Forests. 116 intellectuals including both regional and international scientists as well as trainees gathered at the Colorado State University Mountain Campus for this three-day forum. Current trends in virology and prion disease research were discussed both in talks and poster presentations. This year’s keynote address emphasized innate immune modulation by arboviruses while other invited speakers shared updates on noroviruses, retroviruses, coronaviruses and prion diversity. Additionally, the need for and importance of better approaches for sharing science with non-science communities via science communication was discussed. Trainees and junior investigators presented 19 talks and 31 posters. This report encapsulates selected studies presented at the 22nd Rocky Mountain National Virology Association meeting held on 30 September–2 October 2022.

## 1. Introduction

With the aim of providing a platform for regional scientists and scholars to exchange knowledge on advances in virology, the Rocky Mountain Virology Club (RMVC) was established in 2000. RMVC also focused on providing graduate students with the opportunity of meeting senior scientists thereby nurturing healthy collaborations between intellectuals in the field. In 2009, passing a milestone in the journey, the RMVC expanded the team and scientific boundaries by adding prion biologists to the group. The following year, RMVC was renamed as Rocky Mountain Virology Association (RMVA). Since the first meeting in 2001 it has become customary to have the annual RMVA meeting at the Colorado State University Mountain Campus and the number of participants has increased gradually during the past twenty-two years.

**Figure 1:**
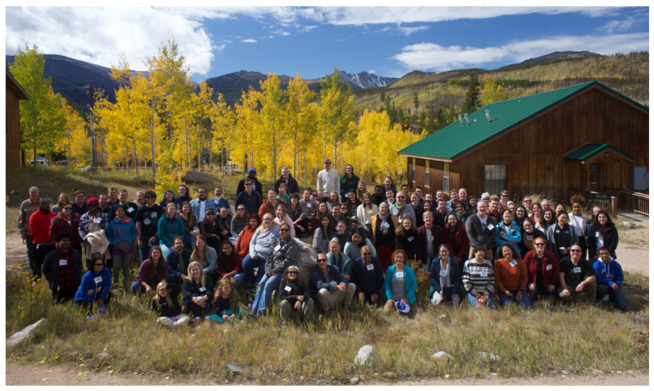
Attendees at the 22nd Rocky Mountain Virology Association conference.

As in previous years, the gathering this year consisted of attendees including undergraduate and graduate students, postdoctoral fellows, research scientists and junior and senior faculty members accounting for a total of 116 participants (Figure 1). The meeting was more vibrant in the presence of scholars coming from varying disciplines in virology, prion biology, immunology and pathology. The three-day forum was split between five sessions, seven invited speakers, 19 oral presentations and 32 poster presentations.

The keynote address by Dr. Ana Fernandez-Sesma (Professor of Microbiology, Icahn School of Medicine at Mount Sinai) discussed the ability of arboviruses to modulate innate immune responses to arboviruses. Invited speakers in 2022 included regional scholars as well as international scientists representing various aspects of virology, immunology, prion biology and scientific communication. Dr. Daisy Leung (Associate Professor, Washington University, School of Medicine), Dr. Jason Mackenzie (Doherty Institute, University of Melbourne, Australia), Dr. Jason Bartz (Professor, Department of Medical Microbiology & Immunology, Creighton University), Dr. Chioma Okeoma (Professor, Department of Pathology, Microbiology & Immunology, New York Medical College), Joe Palca (Science Correspondent, National Public Radio) and Bart Haagmans (Department of Virosciences, Erasmus University Medical Center) were this year’s invited speakers. Their presentations included discussions on the molecular basis of nonstructural viral protein functions, identification of alternative viral release mechanisms and antiviral targets, the potential existence of quasispecies of prions, effective science communication, the role of extracellular vesicles (EVs) in the central nervous system during infection and SARS-CoV-2 phenotypic evaluation to better understand emerging variants.

The RMVA meeting has always been a convivial gathering enabling positive interactions among the attendees to better communicate their research. As part of this, participants engaged in the “longest coherent sentence” contest. The aim of the contest was to construct a comprehensive sentence using a word cloud generated with words from all the abstracts presented this year. In addition, one-minute lightning talks presented in a diversity of formats including dance, song, poetry, art or slides by poster presenters evoked significant excitement and amusement. To add to the uniqueness of the meeting, RMVA conducts ‘Fireside chats’ with the keynote speaker as a way of encouraging trainees to engage with invited speakers. Following years of practice, fireside chats and mentoring sessions were conducted on two evenings of the meeting where students had the opportunity to interact with senior scientists in the field to have questioned answered, obtain career guidance, and establish potential collaborations.

**Figure 2:**
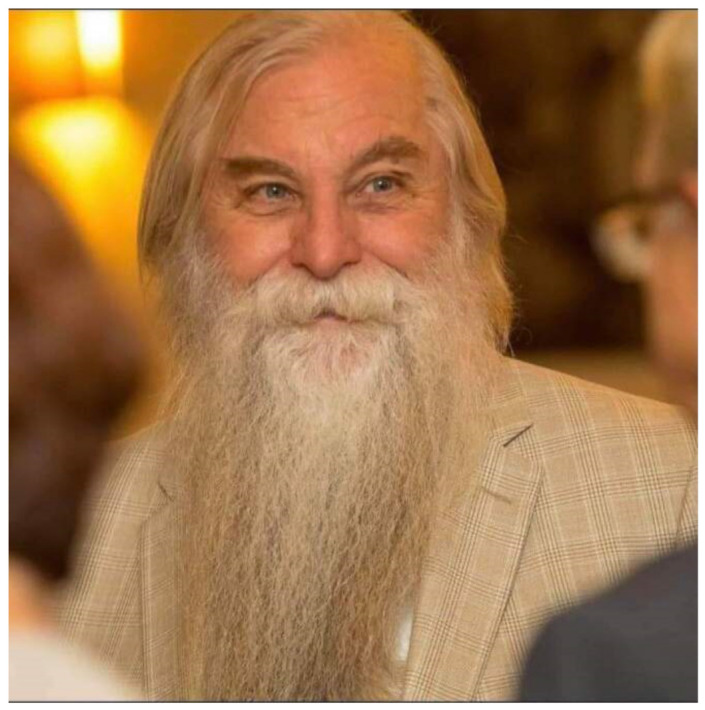
Dr. Randall J. Cohrs. Photo permitted for use by the Cohrs family.

Amongst the intellectually stimulating and impactful sessions of the meeting, the name of Dr Randall J. Cohrs was remembered with reverence (Figure 2). Apart from being a founding member and the president of RMVA, Randy is memorialized as a dear friend, colleague and a great mentor by those who were privileged to associate with him. Even after a year of his passing, the RMVA cherishes his indelible service to our virology community. For those of us who did not meet Randy, there were a number of fond memories shared about him and it was evident that the absence of Dr. Cohrs was felt deeply at this year’s conference. His contribution towards establishing countless global collaborations was immense and he was dedicated to ensuring that students and junior scientists were given ample opportunities to share knowledge via regional, national and international conferences. He is admired for his kindness, magnanimity and friendship and will be dearly missed for his remarkable passion towards science and nurturing younger scientists. In recognition of the magnanimous service by Dr. Cohrs, the Randall, J. Cohrs Awards Fund was established in 2021 which supports the top student and post-doc poster and presentation awards as well as Randall J. Cohrs Keynote address. We are grateful for his initiatives in establishing RMVA and his legacy will be continued through this conference to foster younger scientists.

This is a compendium of presented abstracts at the 22nd Annual meeting of the Rocky Mountain Virology Association.

## 2. Summary of Scientific Sessions

### 2.1. The Randall Jay Cohrs Lecture—Keynote Address

Dr. Ana Fernandez-Sesma (Department of Microbiology, Icahn School of Medicine) delivered the keynote address where she presented her labs’ research on the modulation of innate immune responses by arboviruses. Dengue virus (DENV) belongs to the *Flaviviridae* family and is endemic in more than 100 countries in the world. There are 4 different serotypes that co-circulate and can cause annual epidemics in tropical and subtropical areas of the world and are transmitted by *aedes.sp* mosquitoes. They, and others, showed that DENV can efficiently inhibit the generation of innate immune responses in infected cells by blocking both the production and signaling of type I interferons (IFN) in susceptible cells, including dendritic cells (DCs). They found that the DENV protease complex (NS2B3) can mediate the evasion of innate immunity in those cells and others by targeting and inducing the degradation of different innate immune factors, including cGAS and STING, which results in the inhibition of type I IFN production and antiviral responses. Moreover, they showed that leakage of mitochondrial DNA (mtDNA) induced during DENV infection can trigger the activation of the cGAS/STING pathway of type I IFN production. This mechanism of immune activation triggered by danger signals rather than viral RNA is the focus of their research. They have also reported that different DENV serotypes can induce different immune profiles in infected primary cells and that infected and bystander cells both contribute to the overall immune response in DENV infection by secreting different cytokines and chemokines. Their multi-dimensional analysis of dendritic cells infected with DENV-2 or DENV-4 revealed distinct infection kinetics and immune profiles in DCs that may contribute to the pathogenesis and transmission of these viruses. Additionally, they will discuss how other arboviruses, such as Chikungunya virus (CHIKV) share these innate immune modulatory strategies. All animal studies were performed following guidelines and protocols approved by the Institutional Animal Care and Use Committee of Icahn School of Medicine at Mount Sinai. All studies using human subjects or tissue samples have been either approved or deemed non-human subject research by the Institutional Review board of Icahn School of Medicine at Mount Sinai.

### 2.2. Mechanisms of Virus–Host Interactions

Daisy W. Leung from the Department of Medicine at Washington University in St., Louis spoke about the molecular basis for RSV nonstructural protein functions. Human respiratory syncytial virus (hRSV) is a significant cause of lower respiratory tract infections in children under the age of 5, the elderly, and immunocompromised individuals, leading to millions of hospitalizations that also contribute to mortality. Prior exposure to hRSV affords little protection for subsequent infections and therefore individuals are susceptible to reinfection. Despite the substantial burden on global human health, there are limited effective and safe prophylactic and therapeutic options available. The hRSV genome encodes for two ORFs at the 3′ end, the multifunctional nonstructural proteins NS1 and NS2. These proteins are implicated in several functions, including immune antagonism. However, the mechanisms by which NS1 and NS2 proteins modulate host responses remain incompletely defined. They recently solved the X-ray crystal structures of NS1 and NS2. These structures provide the molecular basis for function and have advanced our understanding of nonstructural protein interactions with host components. Consequences of host-viral interactions include direct inhibition of cytosolic pathogen sensors and binding to host nuclear transcriptional machinery to shape the immune response to infection. Insights from these studies also provide a basis to target NS1 and NS2 therapeutically, including opportunities to generate structure-guided mutant viruses as future vaccine candidates with altered host interactions through NS1 and NS2. No animal or human studies were performed. This work was supported by the NIH (R01AI107056, R01AI140758, and P01AI120943) and by the Children’s Discovery Institute (PDII2018702).

Seth Frietze, with Donna Neumann and David Bloom (Department of Biomedical and Health Sciences, University of Vermont), shared their research on spatial positioning of the latent HSV-1 genome in human neurons. The spatial positioning of genetic loci within the nucleus is a fundamental determinant of gene expression. Human alphaherpesviruses establish a lifelong latent infection in the nucleus of host sensory neurons where viral genomes persist as chromatinized episomes. While alphaherpesvirus latency is known to interact with repressive host structures including heterochromatic posttranslational histone modifications, the role of higher-order chromatin structures in latency and reactivation remains poorly understood. Here, they developed chromatin confirmation capture assays to explore virus chromatin structures and virus–host chromatin interactions during primary and latent infections in human cell models. They further applied capture HiC analysis to map the genome-wide chromatin contacts of latent and reactivating HSV-1 in human neurons. Overall, these studies will offer a deep understanding of the subnuclear host–virus chromatin interactions and will provide technical insight into studying the epigenetic mechanisms of human herpesviruses. No animal or human studies were performed, and the study was Supported by NIH-NIAID R01134807.

Deepashri Rao, along with Kimberly Meade-White, Elaine Haddock, Heinrich Feldmann and David Hawman from the Laboratory of Virology at Rocky Mountain Labs shared their research on innate immune correlates of protection in mouse-adapted Crimean-Congo hemorrhagic fever infection. The widely distributed tick-borne Crimean-Congo hemorrhagic fever virus (CCHFV) causes severe febrile illness in humans. How the host senses and controls the infection and whether host responses contribute to disease is unclear. They developed a mouse-adapted strain of CCHFV that causes significant clinical disease in adult wild-type (WT) mice characterized by 20% weight loss, ruffled fur, and hunched posture and with an additional sex-linked difference in virulence with male mice demonstrating more severe disease than female mice. Worse disease in male mice has many similar correlates to worse disease in humans with male mice having higher viral loads, more severe tissue pathology and greater inflammatory cytokine production. These distinct disease outcomes provide a model to examine whether distinct host responses mediate disease severity upon infection with CCHFV. Deepa and group hypothesize that early innate immune responses to CCHFV infection dictate disease severity and outcome and that early proinflammatory cytokines may contribute to severe disease. They found that therapeutic blockade of proinflammatory cytokines CCL2 or TNFα in infected WT mice did not improve disease. In support, infection of CCL2 or TNFα receptor KO mice also did not show improved disease. Future studies are directed at understanding the role of TNFα and TNF receptors in CCHFV infection, and how they may account for the differential disease severity observed in mice. The study was funded by the Intramural Research Program, NIAID. All animal studies were performed following guidelines and protocols approved by the Rocky Mountain Laboratories Institutional Animal Care and Use Committee.

JA Westrich, EE McNulty, M Carpenter, M Burton, A Sandoval, C Mayo, and CK Mathiason from the Department of Microbiology, Immunology, and Pathology at Colorado State University presented their research on determining longitudinal viral progression and immunological responses to Bluetongue Virus in experimentally infected ruminants. Bluetongue virus (BTV) is an economically important arthropod-borne pathogen that infects ruminant species worldwide. The severity of BTV infections ranges from asymptomatic to lethal, with the most severe cases succumbing to disease within one week. Animals that survive the infection often require months to fully recover. The immune response to BTV infection is thought to contribute to both the propagation of disease as well as being critical in the ultimate resolution of infection. Although BTV has been recognized since the 1800s, much of the cellular and cytokine response remains poorly understood due to limited reagent availability for the natural host species. To gain a greater understanding of the role the immune response plays in BTV infection, they infected cohorts of sheep and muntjac with two different strains of BTV, BTV-10 and BTV-17. Interestingly, the two serotypes showed highly similar progression between the inoculated cohorts. Viremia was monitored by RT-qPCR using BTV-specific primers on intermittent blood draws. Immune cells and cytokines were evaluated by traditional flow cytometry, RNA flow cytometry, RT-qPCR, and/or fluorescent-based antibody arrays. All BTV-inoculated animals exhibited clinical signs characteristic of BTV disease with some potential species-specific differences, specifically in the timing of immune response and viral titers. Circulating virus was observed as early as 3 days post-inoculation (dpi) and remained detectable for the remainder of the study (24 dpi). A distinct pan-leukopenia was observed between 8–14 dpi that rebounded to mock-inoculated control levels at 17 dpi. We observed increased expression of pro-inflammatory cytokines after 8 dpi, notably the pro-inflammatory cytokine CXCL10. Taken together, they have established a model of BTV infection in two separate ruminant species and successfully monitored the longitudinal immunological response and viral progression using a combination of traditional methods and cutting-edge technology. All animal studies were performed following guidelines and protocols approved by the Institutional Animal Care and Use Committee of Colorado State University and the study was funded by USDA-NIFA AFRI grant # 2019-67015-28982 as part of the joint USDA-NSF-NIH-BBSRC-BSF Ecology and Evolution of Infectious Diseases program as well as by grants from the Colorado Research Council of the College of Veterinary Medicine and Biomedical Sciences from Colorado State University and Department of Microbiology, Immunology, and Pathology.

Jason Mackenzie shared research from his lab and collaborators, Joshua Deerain, Turgut Aktepe, Svenja Fritzlar, Katelyn Charry (Department of Microbiology and Immunology, University of Melbourne), Jaclyn Pearson (Centre for Innate Immunity and Infectious Diseases, Hudson Institute of Medical Research), and Peter White (School of Biotechnology and Biomolecular Sciences, University of New South Wales) on norovirus, entitled “Norovirus: Live and let die”. Human norovirus is the leading cause of acute gastroenteritis worldwide with ~685 million cases and over 200,000 deaths annually. Despite the significance of this pathogen, we have a limited understanding of how noroviruses infect, cause disease, and modulate the innate immune response. Programmed cell death (PCD) is an important part of the innate response to invading pathogens, but little is known about how specific PCD pathways contribute to norovirus replication and facilitate clearance and inflammation. Here, they reveal that murine norovirus (MNV) virus-induced PCD in bone marrow-derived macrophages correlates with the release of infectious virus. They subsequently show, genetically and chemically, that MNV-induced cell death and viral replication occur independent of the activity of inflammatory caspase-1, -11 and -12, extrinsic RIPK1, RIPK3 and caspase-8, and pyroptotic effector gasdermin D. Further analysis revealed that MNV infection promotes the cleavage of apoptotic caspase-3 and PARP. Correspondingly, pan-caspase inhibition, or BAX and BAK deficiency, perturbed viral replication rates and delayed virus release and cell death, confirming that MNV infection promotes intrinsic apoptosis to facilitate viral replication and dissemination. In addition, they have linked the functional capacity of the MNV NS3 protein to attenuate protein translation with the loss of pro-survival proteins MCL-1, BCL-XL and BCL-2. Via a mutagenesis approach, they have mapped the domain within the NS3 protein that promotes host protein shut-down and apoptosis induction. Overall, these studies provide an alternative virus release mechanism for norovirus and also a potential target for antiviral drug development. This research was supported by the National Health and Medical Research Council (NHMRC) of Australia. No animal or human studies were performed and the harvesting of primary murine cells was performed following the guidelines and protocols approved by the Animal Ethics Committee of The University of Melbourne.

Monica Graham ^1^ and Benjamin Akiyama ^2^, Camille Merrick ^1^, Matthew Szucs ^1,2^, Jeffrey Kieft ^2^, and David Beckham ^1^ (^1^ Department of Immunology & Microbiology, University of Colorado Anschutz Medical Campus, ^2^ Department of Biochemistry and Molecular Genetics, University of Colorado Anschutz Medical Campus) discussed that Zika virus DB-1 structure is critical for sub-genomic flaviviral RNA formation and virus cytopathic effect during infection. Zika virus (ZIKV) contains multiple conserved RNA structures in the viral 3′ untranslated region (UTR), including the structure known as dumbbell-1 (DB-1). Research suggests the DB-1 structure is important for flavivirus genome replication and cytopathic effect (CPE). However, it has not investigated how the highly conserved DB-1 structure is important for its function. To investigate the DB-1 structure-function relationship, they created two mutant infectious ZIKV clones: TL.PK, which disrupts DB-1 tertiary folding; and p.2.5′, which alters DB-1 secondary structure formation. In A549 cells, viral genome replication of both mutant clones is not significantly reduced compared to ZIKV-WT, but viral CPE is. They investigated sub-genomic flaviviral RNA (sfRNA) formation by both DB-1 mutants following A549 infection and found they both produce less of all sfRNA species compared to ZIKV-WT. To investigate the mechanism of decreased CPE in their mutant isolates, they assayed mutant-infected A549 cells for cell viability and caspase activation. They found that cell viability is significantly increased in DB-1 mutant-infected cells compared to ZIKV-WT. They also determined that cells infected with either mutant clone have decreased levels of activated caspase 3 but not caspase 1 during infection. Curiously, they found that replication of our DB-1 mutants is not restricted by Type I interferon treatment, nor does mutant infection lead to a significant change in Type I interferon-stimulated gene expression compared to ZIKV-WT. They in vitro transcribed sfRNAs from ZIKV-WT and transfected them into DB-1 mutant-infected cells. Finally, in vivo studies in AG129 mice show that mice infected with ZIKV-TL.PK or p.2.5′ survived almost a week longer than mice infected with ZIKV-WT. Overall, data from their TL.PK and p.2.5′ isolates indicate that the ZIKV DB-1 structure is important for sfRNA formation, cytopathic effect, and pathogenesis of ZIKV. All animal studies were performed following guidelines and protocols approved by the Institutional Animal Care and Use Committee of the University of Colorado Anschutz Medical Campus. The study was funded by NIAID/1R01AI153724-01A1; ARMY/W81XWH-17-1-0183

Oshani Ratnayake ^1^ along with Camryn S Guenther ^1^, Elena Lian ^1^, Paul S. Soma ^1^, Laura St Clair ^1^, Rebekah Gullberg ^1^, Angel Balmaseda ^2,3^, Jason M. Mackenzie ^4^, Eva Harris ^5^, Rushika Perera ^1^ (^1^ Center for Vector-borne Infectious Diseases, Dept. of Microbiology, Immunology, and Pathology, Colorado State University, ^2^ Sustainable Sciences Institute, Managua, Nicaragua, ^3^ Laboratorio Nacional de Virología, Centro Nacional de Diagnóstico y Referencia, Ministry of Health, Managua, Nicaragua, ^4^ Doherty Institute, University of Melbourne, Australia,^5^ Division of Infectious Diseases and Vaccinology, School of Public Health, University of California, Berkeley, Berkeley, CA, USA) presented on investigating the role of phospholipase A2 and its metabolites during flavivirus infection of human hosts. Dengue viruses (DENVs) are transmitted by *Aedes aegypti* mosquitoes causing over 400 million infections annually. Upon infection, these viruses hijack host lipid metabolic pathways to support their replication. Specifically, they and others have shown that de novo lipid biosynthesis is actively upregulated via viral protein NS3 to support the formation of replication factories on modified membranes. Interestingly, they have shown that metabolites arising from the opposing pathway, lipolysis, are also altered during infection of both human and mosquito hosts. In the human host, inhibition of lipolysis disrupted viral replication. Analysis of serum isolated from dengue patients also show an enrichment of lipolytic metabolites specifically in patients with severe disease. In the mosquito host, lipolytic metabolites were elevated during infection, prominently differentiating between infections with DENVs as well as Chikungunya, and Zika viruses.

The activity of cytosolic and secreted phospholipase A2 enzymes (PLA2s), releases arachidonic acid (AA) and lysophospholipids from cell membrane phospholipids by the Lands cycle. AA is a well-known precursor of anti-viral inflammatory mediators. Previous studies have shown that reduction in AA and lysophospholipids through a knockdown of AA-producing PLA2 isoforms (2A, 4A, and 4C) decreased DENV2 infectious viral titer. In the current study, they have investigated the specific steps of the viral lifecycle that are impacted by the reduction in AA and lysophospholipids using the loss of function studies of selected PLA2 enzymes. Further, the effect of exogenous AA addition on viral infection and rescue of infectious virus production in PLA2 knockdown cells were investigated. They hypothesized that PLA2 and AA may have dual roles during virus infection, both in supporting viral replication and creating the anti-viral inflammatory precursor molecules. All studies using human subjects or tissue samples have been approved by the Institutional Review Board of Colorado State University. The study is funded by NIH/NIAID grant number RO1AI151166-01 and The Boettcher foundation Early Career Investigator Award.

Chioma M. Okeoma ^1,4,^* along with Hussein Kaddour ^1,2^, Marina McDew-White ^3^, Miguel M. Madeira ^1^, Malik A. Tranquille ^1^, Stella E. Tsirka ^1^, Mahesh Mohan ^3^ (^1^ Department of Pharmacology, Stony Brook University Renaissance School of Medicine, ^2^ Regeneron Pharmaceuticals, Inc., ^3^ Host Pathogen Interaction Program, Southwest National Primate Research Center, Texas Biomedical Research Institute, ^4^ Department of Pathology, Microbiology, & Immunology, New York Medical College) shared research on SIV and Δ ^9^-THC induced alterations in host miRNAome: insights from extracellular vesicles. HIV infection of the central nervous system (CNS) impairs the integrity of the basal ganglia (BG) and is associated with inflammation-related increase in BG white matter and secretion of neuromodulatory host factors that dysregulate CNS cells (neurons, microglia, and astrocytes). How HIV-induced neuromodulatory molecules bring about CNS dysfunction is unclear but may be mediated by extracellular vesicles (EVs). Although the BG is a major target and reservoir of HIV in the CNS, whether BG produces EVs and the effect of HIV and/or the anti-inflammatory phytocannabinoid―delta-9-tetrahydrocannabinol (THC) on BG-EVs remain unknown. Moreover, direct in vivo evidence of the presence of neuromodulatory molecules in BG-EVs and their ability to alter the functions of CNS cells are yet to be determined. These questions were investigated in this study. A total of nine age and weight-matched pathogen-free male Indian Rhesus Macaques (RMs) were randomly assigned to three experimental groups. Group 1 (*n* = 3) received twice daily injections of vehicle (SIV). Group 2 (*n* = 3) received twice-daily injections of THC (THC/SIV) started four weeks prior to SIV infection until 5 months post-SIV infection. Group 3 (*n* = 3) served as uninfected controls (VEH). They obtained BG tissues from the brain of all RMs at necropsy for isolation of EVs. The EVs were subjected to small RNA sequencing for analysis of EV cargo. They also used primary astrocytes from the brains of mice to investigate the functions of EVs. EVs are present in BGs (BG-EVs). There were no significant differences in the physical properties of BG-EVs across the groups. However, SIV infection and THC treatment are associated with significant changes in BG-EV-associated miRNA. BG-EVs from SIV-infected RMs contained 11 significantly downregulated miRNAs. Remarkably, treatment with THC is associated with significant upregulation of 37 miRNAs (including the 11 downregulated miRNA) in BG-EVs. Most of these miRNAs are predicted to regulate inflammation/immune regulation, TLR signaling, Neurotrophin TRK receptor signaling, and cell death/response pathways. Furthermore, BG-EVs were internalized by primary mouse astrocytes, increased the expression of astrocyte marker GFAP, and CD40, TNFα, MMP2, and MMP9 gene products. Their findings reveal a role for BG-EVs as a vehicle with the potential to disseminate HIV and THC-induced changes within the CNS. All animal studies were performed following guidelines and protocols approved by the Institutional Animal Care and Use Committee of SUNY Stony Brook, School of Medicine and Texas Biomedical Research Institute. This work was supported by National Institutes of Health funding (Grant No. R01DA042348 [to CMO]; Grant Nos. R01DA042524 and R01 DA052845 [MM], Grant Nos. R01DA050169 and R21/R33DA053643 [to CMO & MM]; and Grant Nos. R01MH123093 and R01DA029718 [SET] and R01MH123093S1 [MMM].

Obaiah Dirasantha ^1^, along with W Fattor ^1^, C Warren ^1^, N Meyerson ^1^, KW Gregory ^2^ and SL Sawyer ^1^ (BioFrontiers Institute, Department of Molecular, Cellular, and Developmental Biology, University of Colorado at Boulder ^1^ and Department of Veterinary Sciences, North Carolina State University ^2^) discussed the role of baboon TRIM5α proteins in protecting baboons against infection by simian immunodeficiency viruses (SIVs). Simian immunodeficiency viruses (SIVs) comprise a group of retroviruses that cause persistent infection in over 40 different species of African nonhuman primates. Baboons, which are also native to Africa and share their habitat with other SIV-infected nonhuman primates, are not known to persistently harbor any SIV infection. The reasons for this unusual natural resistance to SIV infections in baboons remain a long-standing mystery. One explanation may be that baboons encode a potent restriction factor that exerts broad anti-SIV effects. The alpha isoform of the tripartite motif-containing protein 5 (TRIM5α) is a potent retrovirus restriction factor. To determine whether or not baboon TRIM5α has broad activity against diverse SIVs, they have sequenced and cloned 10 unique TRIM5α alleles derived from a population of 213 captive baboons. They are currently exploring the breadth of baboon TRIM5α activity against a diverse panel of SIV/HIV viruses. Their findings have the potential to uncover mechanisms of natural host resistance to SIV infection, which could be valuable in developing antiretroviral therapies. All animal studies were performed following guidelines and protocols approved by the Institutional Animal Care and Use Committee of the University of Colorado Boulder. The study was funded by NIH-NIDA

Paul S. Soma ^1^, along with Rebekah C. Gulberg, M. Nurul Islam ^1^, Angel Balmaseda ^2,3^, Carol D. Blair ^1^, Barry J. Beaty ^1^, John T. Belisle ^1^, Eva Harris ^4^ and Rushika Perera ^1^ (^1^ Department of Microbiology, Immunology, and Pathology, Colorado State University, Fort Collins, CO, USA; ^2^ Sustainable Sciences Institute, Managua, Nicaragua; ^3^ Laboratorio Nacional de Virología, Centro Nacional de Diagnóstico y Referencia, Ministry of Health, Managua, Nicaragua; ^4^ Division of Infectious Diseases and Vaccinology, School of Public Health, University of California, Berkeley, Berkeley, CA, USA) presented his and his colleagues work on perturbed serum metabolome in dengue infection and disease. Dengue viruses (DENVs) place 3.9 billion people at risk of infection each year, rendering them the most prevalent arboviruses worldwide. These viruses are the etiologic agents of dengue fever (DF) which is a self-limiting disease, but some cases progress to dengue hemorrhagic fever/dengue shock syndrome (DHF/DSS) with vascular leakage leading to shock and potentially death. Severe manifestations present within 4–7 days of fever onset, and reliable biomarkers that predict this progression are urgently needed. A dynamic metabolic response to DENV infection and disease can be measured in human serum, presenting a viable option for dengue disease biomarker detection and insights into the biology of dengue disease severity.

Serum samples from 535 Nicaraguan children (1–14 years) were collected during days 1–7 of illness. Clinical metadata included disease state, sex, virus serotype, and infection history. The serum metabolome was measured using liquid chromatography-mass spectrometry-based untargeted metabolomics that revealed 3807 dysregulated molecular features across disease states. To unlock the underlying metabolism and biology within this dataset, pathway analysis was performed via the mummichog algorithm to glean information on dysregulated metabolic pathways. Thus far, 32 metabolites have been identified (MSI level 1) that are involved in the metabolic pathways of tryptophan, arachidonic acid, omega-3 fatty acids, fatty acid biosynthesis, purines, small peptides, amino acid, carnitines, lysoglycerophospholipids and bile acids. Dysregulation of identified and putatively identified serum metabolites can be used to make inferences about the biology and progression of dengue disease. The results reveal that the serum metabolome relates to dengue disease state and can be used to identify novel, and recapitulate known, metabolic shifts. This work was funded by R21 R33 AI100186 NIH-NIAID. All studies using human subjects or tissue samples have been approved by the Institutional Review Board of Colorado State University.

Phillida A. Charley, along with Kira W. Douglas, and Tony Schountz (Department of Microbiology, Immunology, and Pathology; Colorado State University, Fort Collins, CO, USA) discussed her research on Innate Immune Responses of Bat Cells Infected with Middle Eastern Respiratory Syndrome Coronavirus. The Middle East Respiratory syndrome coronavirus (MERS-CoV) is a betacoronavirus that causes Middle East respiratory syndrome in humans. Bats are believed to be a natural reservoir because they are hosts for a variety of related coronaviruses. Thousands of coronavirus sequences have been identified in bat species spanning Asia, Africa, the Americas, and Europe; however, there are limited data on the interactions between host immunity and the virus. Previous studies have determined that deletion of MERS-CoV genes encoding for proteins 4a and 4b enhances the interferon response in infected human cell lines compared to wildtype MERS-CoV. This study aims to determine what immune pathways are used by Jamaican fruit bats (*Artibeus jamaicensis*) when infected with wildtype MERS-CoV or the Δ4ab deletion mutant. We have determined that Jamaican fruit bat kidney cell lines are susceptible to MERS-CoV and the Δ4ab mutant. Several genes have increased in the MERS-CoV Δ4ab but are lower in the WT infection. Furthermore, MERS-CoV WT and Δ4ab cause more cytopathic effects after ten viral passages. Sequencing data on viral passage show various mutations throughout the MERS-CoV genome. This work was supported by the National Institute of Allergy and Infectious diseases under grant RO1A1140442. No animal or human studies were performed.

Logan Dean ^1^, along with Rebekah C. Gullberg ^1^, Laura St. Clair ^1^, Claire Huang ^2^, and Rushika Perera ^1^ (^1^ Dept. of Microbiology, Immunology, and Pathology, Colorado State University. ^2^ Arboviral Diseases Branch, Division of Vector-Borne Diseases, Centers for Disease Control and Prevention) shared his research on strand-specific quantification of dengue virus, serotype 2 RNA following inhibition of lipid and purine metabolism. Flaviviruses infect over 400 million people globally each year. Vaccination and therapeutic drug development have been unsuccessful and alternative therapeutic targets are needed. Dean and colleagues have previously shown that disruption of fatty acid (FAS) metabolism is a potent antiviral mechanism specifically for flaviviruses. To accurately determine the effects of FAS-disrupting therapeutics on viral replication, they carried out strand-specific quantification of dengue virus, serotype 2 (DENV2) positive sense (genomic) and negative sense (replicative) RNA. They observed significant decreases in viral RNA quantities, both genomic and replicative, following treatment with FAS inhibitors Orlistat and C75. In addition, as their previous work has identified a compensatory use of purines following FAS inhibition of DENV2-infected cells, they evaluated the impact of combinatorial inhibitors that disrupted both FAS and purine metabolism. They demonstrate a reduction in genome amplification following combinatorial therapy, proving this assay allows for accurate observation of the direct impact of therapeutics on viral genome replication. This was work was funded by startup funding from the Department of Microbiology, Immunology and Pathology and the Boettcher Foundation Early Career Investigator Award. The findings and conclusions in this report are those of the authors and do not necessarily represent the official position of CDC. No animal or human studies were performed.

Suad Elmegerhi ^1^, along with Elena Lian ^1^, Gabriela Ramirez ^1^, Carley McAlister ^1^, Brielle Patlin ^2^, Hadley Gary ^1^, St. Clair ^1^, Camryn S Guenther ^1^, Rupika Delgoda ^3^, Stu Tobet ^2^, Brendan Podell ^1^ and Rushika Perera ^1^ (^1^ Center for Vector-borne Infectious Diseases, Department of Microbiology, Immunology, and Pathology, Colorado State University, Fort Collins, CO; ^2^ Department of Biomedical Sciences, Colorado State University, Fort Collins, CO; ^3^ Natural Products Institute, University of the West Indies, Mona, Jamaica) shared her group’s research on utilizing novel platforms and metabolic synergy for evaluating therapeutic interventions for viruses with the potential to cause an epidemic. The current pandemic exposed a need for effective and readily accessible antivirals. They can improve disease outcomes and reduce disease severity as well as continuous viral transmission which lead to new virus variants and frequent waves of infections. Towards solving these issues, Suad and her colleagues have developed numerous in vitro assays via utilizing different cells, ex vivo platforms such as three-dimensional lung slice models, and in vivo platforms that include the development of animal models that depict a range of metabolic illnesses such as diabetes and obesity. Utilizing these in vitro, ex vivo, and in vivo platforms, they have collected data on a wide range of investigational compounds and drugs.

These platforms are also well-suited for analyzing individual or combinations of drug treatments. Combinatorial therapies are essential because mounting evidence indicates that, despite the possibility of some ameliorating benefits from monotherapies of FDA-approved medications, these treatments fall short of providing effective interventions. Due to variable metabolic interactions, people react differently to monotherapies, leading to idiosyncratic, hyporeactive, hyperreactive, or tachyphylaxis symptoms. To increase therapeutic efficacy, two novel strategies will represent a quantum leap; (i) finding synergistic combination therapies that will enable the use of low-dose interventions with increased efficacy and higher therapeutic indices to expand the number of selective therapies. (ii) understanding the metabolic impact of drugs and their effect on the host’s metabolism led us to understand antiviral mechanisms. Data on these developed platforms were presented. This work was funded by The Office of the Vice President for Research CSU, The Boettcher foundation, and The Anschutz Family Foundation. All animal studies were performed following guidelines and protocols approved by the Institutional Animal Care and Use Committee of Colorado State University.

Imali Kegode ^1,#^ together with Sarah Leach ^1,#^, Monica Graham ^1^, and J. David Beckham ^1,2,3^ (^1^ Department of Immunology and Microbiology, University of Colorado School of Medicine; ^2^ Department of Medicine, Division of Infectious Diseases, University of Colorado School of Medicine; ^3^ Rocky Mountain Regional Veterans Administration, Aurora, CO. ^#^ Both authors contributed equally as co-first authors.) presented their work on defining the role of the Zika virus xrRNA2 structure in viral pathogenesis. Zika virus (ZIKV), like most mosquito-borne flaviviruses, contains two highly conserved 3-dimensional (3D) RNA structures within the 3′untranslated region (UTR) that disrupt 5′ → 3′ host exoribonuclease Xrn1 activity. The disruption of Xrn1 at these 3′UTR RNA structures results in the accumulation of subgenomic flavivirus RNAs (sfRNAs), which have been implicated to support viral pathogenesis through interactions with the host cell. The two 3D RNA structures that interact with Xrn1 are termed exonuclease-resistant RNA1 and RNA2 (xrRNA1 and xrRNA2). While the role of xrRNA1 in sfRNA1 biogenesis and flavivirus pathogenesis has been well characterized, much is still unknown about the role of xrRNA2 during flavivirus pathogenesis. Here, Leach and Kegodi designed a series of point mutations to investigate the role of xrRNA2 in ZIKV sfRNA production and pathogenesis. To develop these mutations, they utilized previous xrRNA1 structural data to inform the introduction of mutations designed to disrupt the 3D ZIKV xrRNA2 structure. They introduced two mutations (C10518G, U10519A) into the ZIKV xrRNA2 structure. The resulting virus, termed L1-R ZIKV, had partially reverted and exhibited unchanged genome replication and pathogenicity when compared to wild-type ZIKV. To stabilize these two mutations, an additional point mutation (C10496G) was inserted into the xrRNA2 structure. The resulting virus containing all three mutations, termed X2.L1 ZIKV, exhibited significantly reduced infectious virion production and reduced viral-induced cell death compared to wild-type ZIKV. Surprisingly, the X2.L1 ZIKV maintained both sfRNA1 and sfRNA2 biogenesis in cell culture. These data show that mutations introduced into the ZIKV xrRNA2 structure result in a significant loss of infectious virions and attenuation despite maintained sfRNA production. Future studies will determine potential RNA-protein interactions at xrRNA2 that may modulate outcomes between positive-strand genome packaging and sfRNA production. This research was funded by NIH/NIAID R01 AI153724, NIH/NINDS R01 NS123431, VA Merit I01BX003863 to J.D.B., and by NIH/NIAID R01 AI133348, NIH/NAID R35 GM118070 to J.S.K. No animal or human studies were performed.

Austin Mejia, along with Gabriela Ramirez and Rushika Perera (Center for Vector-borne Infectious Diseases, Dept. of Microbiology, Immunology, and Pathology, Colorado State University) discussed his research progress on investigating the role of acylglycerolphosphate acyltransferase (AGPAT) pathway during flavivirus infection of human hosts. The geographic range of the mosquitoes *Aedes aegypti* is expanding globally. They can transmit viruses to humans such as dengue (DENVs), Zika, chikungunya, and Yellow Fever. DENV is the most aggressive of these arboviruses with an estimate of 390 million people infected annually. Currently, there are no effective antivirals or effective vaccines for these viruses. DENVs reconfigure the host’s lipid profile for viral replication and immune evasion. Meija seeks to identify novel targets for antiviral development by understanding how these viruses hijack host lipid metabolic pathways for their benefit. DENV particularly alters phospholipids (PL) for viral replication. PL biogenesis depends on the acylglycerolphosphate acyltransferase (AGPAT) pathway. AGPATs transform lysophosphatidic acid into phosphatidic acid. Phosphatidic acid is used to produce PL where AGPAT is a rate-limiting step for PL synthesis. He has shown that AGPATs can reduce or increase the replication of DENVs. He hypothesizes that AGPATs will also alter the replication of other mosquito-borne viruses such as Sindbis virus, an alphavirus also transmitted by mosquitoes. Using the loss of function studies, Meija and colleagues are currently investigating which AGPAT enzymes are required for viral replication. They hope to utilize this information to limit viral replication and transmission. Without an effective vaccine or antiviral, we rely on biocontrol to limit mosquito-borne viruses. Using what Meija et al. learn, they can develop a novel target for biocontrol. No animal or human work was conducted and the study was funded by CSU Office for the Vice President For Research and IMSD Cohort Award.

Samantha M. Pinto and Elayne D. Burshek, Oshani Ratnayake, Camryn S. Guenther, Suad Elmegerhi, Paul S. Soma, Venugopal Pujari, Dean Crick and Rushika Perera (Center for Vector-borne Infectious Diseases, Dept. of Microbiology, Immunology, and Pathology, Colorado State University) presented their work on the role of Triacylglycerol during dengue virus replication in Aedes aegypti. Dengue viruses (DENVs) are flaviviruses, transmitted by the bite of an infected *Aedes aegypti* mosquito. Pinto and Burshek et al. have previously shown that infection of these mosquitoes with DENV, serotype 2 caused significant changes in the lipidome of the mosquito. Further studies using liquid chromatography-mass spectrometry revealed a specific increase in Triacylglycerol (TAG) levels in *Ae. Aegypti* mosquitoes fed with an infectious blood meal containing DENV2. A similar trend was also observed with Zika and Chikungunya virus infections. Pinto and Burshek hypothesize that TAG levels increase in response to virus infection and may play a significant role in viral replication, dissemination and transmission in the vector. Alternately, TAG levels may influence the immune response to infection. To test this hypothesis, they inhibited TAG synthesis as well as TAG hydrolysis in the mosquito host using three chemical inhibitors followed by infection with DENV2. The current study determined the half-maximal inhibitory concentration (IC_50_) of the chemical inhibitor to be used in order to inhibit TAG synthesis without killing the vector. The results of TAG analyses and assays developed was presented. This research was funded by NIH/NIAID grant number RO1AI151166-01 and The Boettcher foundation Early Career Investigator Award. No animal or human studies were conducted.

Molly Ring, along with Paula Lado, Brian Foy (Center for Vector-Borne Infectious Diseases, Department of Microbiology, Immunology and Pathology, Colorado State University. Fort Collins, CO 80521) shared her research on the role of Niemann Pick Type C2 genes during ivermectin blood meal response in mosquito arbovirus and Plasmodium vectors. Endectocide ivermectin (IVM) is being used as a novel vector-borne disease control strategy given its ability to kill mosquitoes and interfere with egg production in surviving mosquitoes. Ring is studying the administration of IVM-dosed bird feed as a method to curb the spread of West Nile Virus and mass drug administration of IVM to humans in West Africa to disrupt the spread of malaria. These strategies depend on drug pharmacokinetics in the treated host, and so mosquitoes invariably will ingest sublethal concentrations depending on when they bite a host relative to the last time they were treated. When mosquitoes ingest a blood meal containing sublethal IVM concentrations, more genes are upregulated than downregulated, and certain classes of upregulated genes predominate, but the roles of many of these IVM-responsive genes are not well understood. Ring has found that members of a specific gene family (Niemann Pick Type C2 family; NPC2) are the most highly upregulated following ingestion of an IVM-containing blood meal. NPC2 traffics cholesterol in vertebrates but may also serve as carriers for other semiochemicals and hydrophobic compounds in arthropods. She used qPCR to measure the transcript levels of two IVM-responsive NPC2 genes AGAP002848 and AGAP002847, and the bloodmeal-responsive NPC2 gene AGAP002851. Transcript levels will be detected in female *Anopheles gambiae* mosquitoes that have been given varying concentrations of IVM-dosed bloodmeals. In future experiments, Ring plans to clone these three transcripts to make dsRNA that can be used for RNAi knockdown assays via intrathoracic injections. She hypothesizes that transcript knockdown of AGAP002848 and AGAP002847 will lead to increased mortality following digestion of an ivermectin spiked blood meal while knockdown of AGAP002851 transcript will lead to sterol transport disruption and ultimately egg production interference. This work was funded by NIH/NIAID grant U01AI138910 and the MIP Undergraduate Research Fellowship. No animal or human studies were performed.

Rubio H, along with Westrich JA, Bettencourt T, McNulty EE, Nalls AV, Mathiason CK (Department of Microbiology, Immunology, and Pathology, Colorado State University) discussed his research on evaluating ZIKA viral proteins capacity for CXCL10 induction in human placental cells. Zika virus (ZIKV) was identified in 1947 in the Ziika forest, Uganda. As a flavivirus, ZIKV is principally spread by mosquito vectors and had a relatively low impact for the first decades after its discovery. During an outbreak in the Americas in 2015, ZIKV was identified to be causally associated with negative birth outcomes, most notably microcephaly. Although ZIKV is capable of crossing the placental barrier, another factor potentially contributing to negative outcomes is the cytokine CXCL10. CXCL10 is an inflammatory cytokine that has pro-apoptotic properties. CXCL10 is highly upregulated in the serum of ZIKV patients with negative birth outcomes and is implicated to cause neuronal apoptosis during fetal development. Currently, the mechanisms of ZIKV-mediated CXCL10 induction remain unknown. Rubio et al. have previously shown that ZIKV infection in human placental cell lines is highly correlated to CXCL10 induction. To evaluate the mechanism of induction, they aim to establish expression plasmid vectors containing the ZIKV proteins. As ZIKV NS5 directly interacts with pathways known to induce CXCL10 they hypothesize this protein is involved. Lastly, different ZIKV strains induce CXCL10 with varying efficacy, thus they aim to determine ZIKV proteins of different strains have different capacities to induce CXCL10. ZIKV continues to circulate across the globe. As climate change continually expands the range of the ZIKV mosquito vector, more humans, including pregnant women, will contract infections that will increase the risk of poor pregnancy outcomes. Thus, studies to determine mechanisms associated with the impact of ZIKV at the maternal-fetal interface are needed to mitigate the negative consequences of future ZIKV infections. Funding for this project was provided by the department of MIP, college of CVMBS CSU, as well as a grant from the Colorado Research Council pilot award. No animal or human studies were performed.

Sanchez-Vargas I, along with Saavedra-Rodriguez K, Moreno-Garcia M, Perera R, Olson K (Department of Microbiology, Immunology and Pathology, Colorado State University) presented research on the association between dengue vector competence and pyrethroid resistance in *Aedes aegypti*. The *Aedes aegypti* mosquito is important for public health because it transmits the dengue (DENV), Zika, and chikungunya viruses to humans. Because there are no effective vaccines to prevent these arboviral infections, disease prevention and control has switched to reducing mosquito populations, principally through the use of pyrethroid insecticides. Unfortunately, its widespread use resulted in the development of pyrethroid resistance in the field. Mutations at the target location, also known as knockdown resistance (*kdr*), and enhanced metabolism are the key mechanisms of pyrethroid resistance in *Ae. aegypti*. Pyrethroid resistance is linked to biological fitness costs such as size, longevity, fecundity, and mating preference. Furthermore, pyrethroid resistance may alter the mosquito’s intrinsic susceptibility to virus infection, reproduction, and transmission, which is commonly referred to as vector competence (VC). Sanchez-Vargas et al. explored if DENV2 VC is associated with pyrethroid resistance in *Ae. aegypti* from Mexico. Infection rates (IR) for DENV2 were greater for insecticide susceptible strains (56%–66%) than for pyrethroid-resistant strains (33%–36%). Furthermore, the strain with the highest level of pyrethroid resistance (50-fold) had a considerably lower mean viral titer (2.79 ± 0.11 log_10_PFU) than the susceptible strains (3.88 ± 0.2 log_10_PFU) (*p*-value 0.001). Both ‘strain’ and ‘*kdr*’ explained the variance in viral titer using a generalized logistic model with a Poisson distribution (*p*-value 0.001). Future research is needed to determine whether the lower VC is the result of causal pathways or if it is a universal phenomenon across all diseases transmitted by *Ae. aegypti* in the field. The study was funded by the grant on “Insecticide Resistance Management to Preserve Pyrethroid Susceptibility in Aedes aegypti” under National Institutes of Health R01AI121211. No animal or human studies were performed.

### 2.3. Developments in Viral Mutation, Replication, Transmission and Epidemiology

Christian Smith along with R. Kading and C.L. Campbell (Department of Microbiology, Immunology and Pathology, Center for Vector-Borne Infectious Diseases, Colorado State University) discussed the expression level identification of host cell signaling transcripts in Rift Valley fever virus (RVFV) infected *Aedes aegypti (Aae)* and *Culex tarsalis (Cxt*). RVFV is a zoonotic mosquito-borne virus that infects livestock and humans. In humans, RVFV causes fever-like symptoms, birth defects, and sometimes death. To better understand how mosquitoes respond to infection, they investigated immune system signaling gene expression at 14 days post-infection (dpi). Alterations to these signaling pathways could affect infection rates in the mosquito vectors, *Aae* and *Cxt*. Expression levels of five transcripts were analyzed: *Armadillo* (*ARM*), *Frizzled2* (*FZ2*), and *Disheveled* (*DSH*), of the Wingless pathway, *Puckered* (*PCK*), of the c-Jun N-terminal Kinase (JNK) pathway, and *Domeless* (*DOME*), of the Janus Kinase/Signal Transducers and Activators of Transcription (JAK/STAT) pathway. Using qPCR, RVFV MP-12 infected mosquitoes were compared to uninfected controls and analyzed using a *t*-test (*p* < 0.05). Linear regression of log2 fold-changes and viral log-copy numbers were performed, at *p*-value < 0.05. For *Aae*, it was found that *DSH*, *PCK*, *ARM*, and *DOME* showed significant expression level differences. For *Cxt*, *FZ2* and *ARM* showed significant differences, while *DSH*, *PCK*, and *DOME* did not. When analyzing linear regression, only *DSH* in *Aae* showed a significant correlation between log2 fold-change in expression levels and adjusted log-copy numbers. *Cxt* are more susceptible to MP-12 viral infection with 100% of the samples having viral RNA present 14 dpi, compared to 61.7% in *Aae*, being a potential reason for the differences seen across transcript expression between the two species. These data suggest RVFV infection results in prolonged changes to cell signaling pathways, occurring differently across the two species. This work was funded by the NIH/NIAID through grant number RO1AI148633. No animal or human studies were performed in this study.

Shijun Zhan ^1^ with Maggie Priore ^1^, Miles Eckley ^1^ and Tony Schountz ^1^ (Department of Microbiology, Immunology, and Pathology, Colorado State University) presented their groups work with Sosuga virus (SOSV) infection of Jamaican fruit bat primary kidney epithelial cells. SOSV was first identified in a biologist with febrile disease returning from the South Sudan/Uganda region. It was subsequently determined that Egyptian fruit bats (Rousettus aegyptiacus) are a reservoir host of the newly discovered pararubulavirus. The lab wanted to determine whether SOSV can infect Jamaican fruit bats (Artibeus jamaicensis) or their cells. Three Jamaican fruit bats were challenged with 103 TCID50 of SOSV. One bat had evidence of infection by qPCR in which the virus was detected in its day three blood and urine. For cell culture experiments, 0.1 MOI of the virus was inoculated onto primary bat kidney cells and was independently passaged on Ajk4 and Ajk6 cells 10 times. By passage 10, the viruses replicated to higher titers. The Ajk4 cell-10x passaged virus caused substantial CPE on both Ajk4 and Ajk6 cells while the Ajk6 cell-10x passaged virus ceased to cause CPE on either cell. Sequencing results showed genetic mutations in hemagglutinin-neuraminidase which may account for such changes and further analysis is ongoing. The results suggest that SOSV is capable of infecting cells from New World fruit bats and has the potential to adapt to infect Jamaican fruit bats. Future studies will further passage SOSV in Jamaican fruit bats and their cells to generate an adapted SOSV for Jamaican fruit bats as a surrogate animal model. All animal studies were performed following guidelines and protocols approved by the Institutional Animal Care and Use Committee of Colorado State University.

Marylee L. Kapunscinski and Mark D. Stenglein (Department of Microbiology, Immunology, and Pathology, College of Veterinary Medicine and Biomedical Sciences, Colorado State University, Fort Collins, CO, USA) discussed using minigenome melees as a novel high throughput method to evaluate reassortment potential between segmented RNA viruses. The emergence of novel reassortant viruses with altered phenotypes can lead to high-consequence pathogens with devastating economic and health impacts. As ecological niches change due to climate change and human travel, the opportunity for viral reassortment has also increased. By identifying viruses that occupy the same geographic areas and are molecularly compatible for reassortment we can better inform outbreak preparedness strategies. The ability of the proteins of one virus to functionally interact with the genome segments of a related virus is directly proportional to their reassortment potential. This molecular inter-compatibility has traditionally been studied using minigenome/replicon assays involving one pair of viruses at a time. While these assays have been informative, they are limited in throughput and cannot distinguish between replication, transcription, and packaging. Here, they developed a system that goes several steps beyond traditional minigenome assays by including molecular barcodes that allow them to track replication, transcription, and packaging using next-generation sequencing as a readout. This allows them to evaluate the molecular compatibility of the proteins of one virus for the segments of a large number of related viruses simultaneously and to track these key steps of the virus life cycle separately. Using these “minigenome melees” in concert with traditional techniques they can answer targeted questions about the molecular compatibility between segmented RNA viruses, using orthobunyaviruses as a prototype. This research was funded by a CRC grant from the College of Veterinary Medicine and Biomedical Sciences at Colorado State University and additional funding from the Colorado State University Office of the Vice President for Research. No animal or human studies were performed.

Ryan Jeep ^1^ along with Liangqun Huang ^1^, James Boehlke ^1^, Christian Sanders ^1^, Carol A Carter ^2^, and Chaoping Chen ^1^ (^1^ Department of Biochemistry and Molecular Biology, Colorado State University, Fort Collins, CO 80523-1870, USA, ^2^ Department of Molecular Genetics and Microbiology, Stony Brook University, Stony Brook, NY 11794-5222, USA) discussed the sensitive quantification of HIV-1 infectivity for drug resistance assessment. HIV protease inhibitor (PI) resistance is an ongoing problem that compromises the treatment efficacy and prognosis of combination antiretroviral therapy (cART). Despite the identification of numerous resistance-associated mutations (RAMs) in the protease (PR) gene and beyond, the precise correlation between PR genotype and PI treatment outcome remains to be established. They reported a novel assay for sensitive infectivity quantification of viral particles in response to varying concentrations of PI. Proviral constructs that express a histone H2B-mRFP reporter, driven by the Nef promoter, were engineered for the identification and quantification of individual infected cells. Infectivity was measured in live U2OS cells through colocalization of mRFP signal with nuclear Hoechst staining and reflected as the number of infected cells per 1000 target cells. A typical virus preparation displayed a sigmoidal curve as a function of virus input normalized to total p24, allowing for sensitive quantification of infectious units over a large range of viral inputs. To determine the utility of their assay for drug resistance quantification, they examined a protease double mutant, V77I/V82T, which was originally identified clinically in a patient experiencing indinavir resistance. Their results revealed that mutant viruses treated with 100–200 nM indinavir were more infectious, i.e., contained more infectious units per equal amount of virus input, than wild-type viruses made under the same conditions. To the best of their knowledge, this assay for the first time recapitulated the clinically observed indinavir resistance of the V77I/V82T double mutant in a laboratory setting at PI concentrations matching those anticipated in the plasma of patients under cART. Additionally, mutant viruses became more susceptible to darunavir inhibition, i.e., mutant viruses were less infectious than the wild-type control per equal amount of virus input. Together, their assay offers a sensitive platform not only to study the role of individual RAMs in drug resistance development but also to guide personalized treatment strategies for patients experiencing PI resistance. No animal or human studies were used in this study.

Lexi H. Keene and Mark D. Stenglein (Dept. of Microbiology, Immunology, and Pathology, Colorado State University) presented on the RNA viral metagenomics in 100-year-old Drosophila melanogaster museum specimens. Paleogenomic sequencing has enabled the reconstruction of extinct genomes and elucidated ancient host-pathogen interactions. Most studies that utilize historic specimens focus on DNA as it is considered to be more stable than RNA but this ignores viral RNA in a historical context. Here, they used samples from museum collections to study the interactions between galbut virus, a common persistent viral infection of flies, and its host *Drosophila melanogaster.* To evaluate RNA stability and recoverability in dried insect specimens, they pinned lab-reared *D. melanogaster* for RNA extraction at various timepoints over 18 months. This showed that although RNA molecules fragment increasingly over time, RNA levels remain surprisingly constant and are sufficient for downstream applications such as RT-qPCR and library preparation. Next, to understand how long galbut virus has been infecting *D. melanogaster* and how its sequence might have changed over the past century, they acquired museum specimens of *D. melanogaster* from collections across the United States and extracted RNA for whole-shotgun RNA sequencing. They found that galbut virus has been a persistent infection in flies for at least 100 years. Broadly, these findings lead them to conclude that RNA is more stable than previously thought and even highly degraded RNA can be used in applications such as sequencing to answer relevant biological questions about the RNA within a specimen. This highlights the need to use this type of specimen to provide a clearer understanding of historic virus–host interactions and virus evolution. This work was funded through the NIH qCMB T32 Fellowship (T32GM132057) and an NSF CAREER Grant (2048214). No vertebrate animal studies were performed.

Kathryn Coffin ^1^ and Lado P ^1^, Leon AS ^1^, Ring M ^1^, Sougue E ^1^, Pugh G ^1^, Gray L ^1^, Parikh S ^2^, Colt M ^2^, Ehrlich H ^2^, Fabrice Somé A ^3^, Roch K ^3^ and Foy BD ^1^. (^1^ Center for Vector-Borne Infectious Diseases, Department of Microbiology, Immunology, and Pathology, Colorado State University, Fort Collins, CO 80521, ^2^ Yale School of Medicine, New Haven, CT, ^3^ Insitut de Recherche en Sciences de la Sante, Bobo Dioulasso, Burkina Faso) discussed the detection of plasmodium species in *Anopheles* vectors in Burkina Faso, Africa to determine the role of *Plasmodium* species in malaria epidemiology. Malaria is a vector-borne disease transmitted by mosquitoes of the genus *Anopheles* caused by *Plasmodium* parasites. It affects individuals world-wide, particularly those within the African region. RIMDAMAL II was a clinical trial designed to determine the efficacy of adding ivermectin mass drug administrations to the standard malaria control measures with the aim of reducing the incidence of uncomplicated malaria episodes in children. The trial occurred in Burkina Faso, one of the countries with the highest malaria incidence, and lasted from 2019 to 2020. During RIMDAMAL II, *Anopheles* mosquitoes were captured, preserved, and sent to their lab for analysis. The majority of mosquitoes were identified as *An. coluzzii, An. funestus* s.s, and *An. gambiae* s.s. Three different *Plasmodium* sporozoites species were detected in head and thorax samples of the mosquitos: *P. falciparum* (78/2477 in *An. gambiae* sl and 6/216 in *An. funestus* complex), *P. ovale* (24/2477 in *An. gambiae* sl, and 14/216 in *An. funestus* complex), and *P. malariae* (10/2477 in *An. gambiae* sl and 6/216 in *An. funestus* complex). Coinfections of *P. ovale* and *P. falciparum* were also detected in *An. gambiae* sl (2/2477). The most prevalent *Plasmodium* species detected in *An. gambiae* sl. mosquitoes was *P. falciparum*. However, in *An. funestus* mosquitoes, a high sporozoite rate of *Plasmodium* minor species (*P. ovale* and *P. malariae*) was observed. The detection of high rates of minor malaria species signifies that malaria epidemiology in Burkina Faso might be more complicated than initially thought. Funding for this work was provided by the NIH-NIAID and special thanks to the IRSS-DRO team for sample collections. No animal or human studies were performed.

Emily Fitzmeyer along with Emily Gallichotte, Michael Young, Kyra Pyron, Marylee Kapuscinski and Gregory Ebel (Dept. of Microbiology, Immunology, and Pathology, Colorado State University) discussed using barcoded West Nile virus (bcWNV) to examine the impact of tissue-associated bottlenecks on virus populations in enzootic and bridge vectors of West Nile virus (WNV)**.** When a mosquito feeds on a WNV-infected host, the virus must move through the midgut, salivary glands and into the saliva: the main bottlenecks associated with infection, dissemination, and transmission. Each bottleneck serves as a barrier to transmission, the severity of which can vary by tissue type and potentially by mosquito species. Arboviruses are frequently maintained in nature by multiple mosquito species, often with varying levels of vector competence. While it is known that bottlenecks stochastically reduce the diversity of virus populations during mosquito infection, it remains unknown whether bottleneck severity is impacted by vector competence. They used molecularly bcWNV to quantitatively measure tissue-associated population bottlenecks in *Culex tarsalis, Culex quinquefasciatus,* and *Aedes aegypti*. In all species, they observed reductions in bcWNV population richness and complexity upon escape from the midgut and entry to the salivary glands and visualized this reduction in diversity in individual mosquitoes. In *Culex* mosquitoes, barcodes present at high frequencies in the bloodmeal were consistently maintained as the dominant barcodes in the midgut. However, dominance in the bloodmeal had no bearing on barcode transmission (i.e., presence in the saliva) in any species. In *Aedes* mosquitoes, the diversity of barcodes identified in the midgut was significantly lower compared to *Culex*. Additionally, *Aedes* mosquitoes subverted the barcode maintenance trend observed in *Culex* midguts. However, population richness and complexity did not differ significantly between the salivary gland and saliva samples from any species. This work indicates that bottleneck severity, post midgut infection, does not differ between their vectors of varying competence, and provides insight into how stochastic pressure influences virus population dynamics during systemic mosquito infection. No vertebrate animal or human studies were performed in this study.

James S. Terry ^1^ along with Kitty Brown ^2^, Corey Broeckling ^2^ and Brian Geiss ^1^ (Department of Microbiology, Immunology and Pathology, Colorado State University ^1^ and Analytical Resources Core, Colorado State University ^2^) presented their work using cross-link mass-spectrometry to define protein interactions in flavivirus replication compartments. During infection, flaviviruses form invaginations in the host cell endoplasmic reticulum (ER), termed replication compartments, as safe havens for viral replication. Here, viral nonstructural proteins assemble into replication complexes that synthesize and cap new viral genomic RNAs used for viral protein expression and virion production. Viral nonstructural proteins and host proteins are known to co-immunoprecipitate with the replication complex, indicating their importance in the formation of the complex, and ultimately the replication compartment. The proper formation of these protein interactions is critical for the efficient operation of the replication process, but we currently do not know the full complement of viral and cellular proteins resident in these replication compartments nor how they interact to protect and promote viral genome replication. Identifying the proteins present in these compartments and how they interact is critical for the development of antivirals and vaccines to help control flavivirus infections. Cross-link mass spectrometry is a powerful technique to identify protein:protein interactions and reveal how proteins associate in intact cellular structures. They are therefore using cross-link mass spectrometry to build a map of host and viral protein:protein interactions within isolated replication compartments to help develop accurate models of these structures. ER microsomes were purified from infected HEK 293F suspension cells by differential gradient ultracentrifugation followed by genetic and proteomic characterization. Non-crosslinked fractions were analyzed to establish microsome protein composition while crosslinked uninfected and infected fractions identified interactions between proteins. Preliminary mapping of interactions in flavivirus-induced replication compartments determined by cross-link mass spectrometry will be determined. This work was supported by National Institutes of Health Grant R01 AI132668 to B. J. G. No animal or human studies were performed

Tyler Starr from the Department of Biochemistry, University of Utah discussed shifting mutational landscapes during SARS-CoV-2 evolution. SARS-CoV-2 has evolved variants with substitutions in the spike receptor-binding domain (RBD) that impact its affinity for the ACE2 receptor and recognition by antibodies. These substitutions could also shape future evolution by modulating the effects of mutations at other sites—a phenomenon called epistasis. To investigate this possibility, he performed deep mutational scans to experimentally measure the effects on ACE2 binding of all single amino-acid mutations in the Wuhan-Hu-1, Alpha, Beta, Delta, Eta, and Omicron BA.1 and BA.2 variant RBDs. Some substitutions, most prominently N501Y, cause epistatic shifts in the effects of mutations at other sites. These epistatic shifts shape subsequent evolutionary change, for example enabling many of the antibody-escape substitutions in the Omicron RBD that otherwise would compromise receptor-binding affinity. These epistatic shifts occur despite high conservation of the overall RBD structure. His data shed light on RBD sequence-function relationships and facilitate the interpretation of ongoing SARS-CoV-2 evolution. This work was supported by NIAID/NIH K99AI66250. No animal or human studies were performed.

Lauren Malsick, Michael Mingroni, and Brian Geiss (Department of Microbiology, Immunology, and Pathology, Colorado State University) described a strategy for investigating how the NSP13 protein in SARS-CoV-2 impacts helicase activity and how this may alter viral replication. SARS-CoV-2 is a single-stranded positive sense RNA virus in the *Coronaviridae* family. Coronaviruses encode a dedicated RNA helicase to unwind viral RNA, which is critical for viral replication. The structure of NSP13 was solved in 2021, and this new structure showed significant similarity with the flavivirus NS3 RNA helicase that has been previously studied by their group, suggesting they may have roughly similar functions. In the West Nile virus NS3 helicase, they determined that two mutations in Motif V (T407A and S411A), which is situated between the ATPase and helicase domains, significantly increased the speed at which the helicase unwinds RNA, reduced rates of viral genome replication, and enhanced cytopathic effect in vitro and mortality in vivo compared to parental viruses. These findings suggested that Motif V may serve as a critical regulator of energy transduction within the enzyme and is important for viral genome replication and pathogenesis. Motif V is also present in NSP13, so they hypothesized that Motif V in the SARS-CoV-2 NSP13 helicase would also alter RNA helicase rates. To test this hypothesis, they have generated mutations in analogous NSP13 residues, Thr532A and Ser535A, to investigate if the translocation speed of the helicase is increased compared to wild-type. They have established protein purification protocols and enzymatic assays to study the effects of these mutations on NSP13 helicase function, and are currently in process of establishing enzymatic rates of wildtype and mutant NSP13 proteins. Ultimately, understanding the role of Motif V in NSP13 helicase function may help in the rational design of broad-range antiviral inhibitors for coronaviruses. Funding was provided by: 5R01AI166050 (NIH/NIAID). No animal or human studies were performed for this work.

Kalani M. Williams ^1^, along with co-authors Natalie R. Wickenkamp ^1^, Emma K. Harris ^1^, Benard Matovu ^2^, Betty Nalikka ^2^, Lillian Nalukenge ^2^, Jack-Michael Mutebi ^2^, Aggrey Siya ^2^, Tanya A. Dewey ^3^, Kevin Castle ^4^, Teddy Nakayiki ^5^, Robert M. Kityo ^2^, and Rebekah C. Kading ^1^ (Dept. of Microbiology, Immunology, and Pathology, Colorado State University ^1^, Zoology, Makerere University ^2^, Biology, Colorado State University ^3^, Four Seasons Veterinary Specialists ^4^, Entomology, Uganda Virus Research Institute ^5^) presented their work on unraveling the spatial ecology of two bat species in Uganda, with a focus on how these species may be reservoirs for emerging viral pathogens. The overwhelming majority of viral emerging infectious diseases have been the result of spillover events from wild or domestic animals. Recently, insectivorous horseshoe bats (*Rhinolophus* spp.) have become known for their association with coronaviruses, including strains closely related to SARS-CoV-2, but they have also been associated with hosting several other important viral families. Meanwhile, filovirus RNA has been detected in Angolan rousette bats (*Lissonycteris angolensis*). It has become increasingly critical to understand the ecology of these reservoir hosts, along with where and how spillover events could occur. For *Rhinolophus* and *Lissonycteris* bats on the African continent, there is currently very little known about their movement patterns. In this study, they used GPS tracking to ascertain locations bats select for foraging within the Mount Elgon region of Uganda and determined whether these locations are closer or further from various landscape features than would be expected if their distribution was random. GPS data were acquired by suturing GPS units onto bats and taking fixes once every hour during periods of low activity and once every six minutes during periods of high activity. Using kernel density algorithms to determine foraging hotspots from the distribution of GPS points, they can compare landscape feature preferences in *Rhinolophus* and *Lissonycteris* bats. As the study progresses and more landscape features are incorporated into our analysis, they will develop an ecological niche model that can be used to predict foraging sites. These sites could be targeted for conservation, as well as monitored for risk of viral spillover. This work was funded by the U.S. Department of Defense, Defense Threat Reduction Agency HDTRA1-19-1-0030. Additionally, this work is supported by the National Science Foundation Graduate Research Fellowship Program under Grant No. 006784. Any opinions, findings, and conclusions or recommendations expressed in this material are those of the author(s) and do not necessarily reflect the views of the National Science Foundation. All animal studies were performed following guidelines and protocols approved by the Institutional Animal Care and Use Committee of Colorado State University.

Natalie Wickenkamp ^1^ together with Kalani Williams ^1^, Emma Harris ^1^, Anna C. Fagre ^1^, Jack-Michael Mutebi ^2^, Benard Matovu ^2^, Lillian Nalukenge ^2^, Betty Nalikka ^2^, Aggrey Siya ^2^, Teddy Nakayiki ^3^, Charity Nassuna ^3^, Leonara Nabatanzi ^3^, John Kayiwa ^3^, Kevin Castle ^4^, Tanya Dewey ^5^, Julius Lutwama ^3^, Robert M. Kityo ^2^, and Rebekah C. Kading ^1^ (^1^ Department of Microbiology, Immunology, and Pathology, Colorado State University, Fort Collins, CO, USA, ^2^ Department of Zoology, Entomology, and Fisheries Sciences, Makerere University, Kampala, Uganda, ^3^ Uganda Virus Research Institute, Entebbe, Uganda, ^4^ Wildlife Veterinary Consulting, Fort Collins, CO, USA, ^5^ Department of Biology, Colorado State University, Fort Collins, CO, USA) presented their work on Ecology and bio-surveillance methods for bats and their viral pathogens in Uganda. Emerging and reemerging viral pathogens present a continual threat to global health security, and often wildlife reservoirs involved in spillover events are left understudied. Substantial work remains to develop sustainable and effective bio-surveillance programs in regions of interest to global health. To strengthen active research on bat ecology and bio-surveillance methods in Uganda, the team developed partnerships between US and Ugandan academic institutions and government agencies to characterize public health risks at the human-bat interface. As for the presenters, In May 2021 seasonal sampling of bat communities residing in cave systems in Eastern Uganda was started. This ongoing research includes the use of global positioning systems (GPS), acoustic monitoring, and infrared camera technologies to describe cave community composition, bat dispersal patterns, and bat interactions with both wild and domestic animal communities. Pathogen screening of bat samples via RT-PCR and next-generation sequencing (NGS) has revealed virus circulation within Rhinolophus bat populations. Camera trap observations provide evidence of cave use by humans and wild animal species implicated in previous spillover events. The group concluded that the preliminary data gathered to date reinforce the importance of ecological research and sustainable bio-surveillance programs for the rapid response to future outbreak events. Further, they mentioned that an additional project aims to evaluate the impact of bat dispersal patterns on seasonal virus distribution, the role of citizen science in bat bio-surveillance, and the human risk of exposure to bat-borne viruses are underway. This research was supported by NIH/NCATS Colorado CTSA Grant Number UL1TR002535 and NIH R21 AI146740. Its contents are the authors’ sole responsibility and do not necessarily represent official NIH views. This work was also supported in part by Animal Health and Disease Grant No. 2021-01/Project Accession No. 1027096 from the USDA National Institute of Food and Agriculture.

Ali L. Brehm and Mark D. Stenglein (Center for Vector-Borne Infectious Disease, Department of Microbiology, Immunology, and Pathology, Colorado State University) described tendencies of galbut virus transmission in relation to the genetic background of the *Drosophila* host, with paternally transmitted virus displaying lower RNA levels. Galbut virus is a trisegmented multipartite partitivirus found in 100% of populations of *Drosophila melanogaster* with 100% biparental vertical transmission rates with no identified negative fitness effects. However, the virus is only found in ~60% of individuals, and there is evidence that some flies are resistant to infection. The full-length RNA-dependent RNA-polymerase segment of galbut virus was recently found to be endogenized in the germ line of European populations of *D. melanogaster*. The authors wanted to test the hypothesis that the galbut virus EVE makes *D. melanogaster* resistant to galbut virus infection. They crossed flies homozygous for the EVE with flies from *Drosophila* Genetic Reference Panel line 517, (DGRP-517), which is susceptible, and quantified viral loads in offspring. Offspring from EVE homozygous mothers and galbut-infected DGRP-517 fathers had lower viral loads than offspring from control crosses, indicating that some element of this genetic background made them relatively resistant to paternally transmitted galbut virus. Maternal transmission produced offspring with higher galbut virus levels than offspring from paternal transmission, regardless of genotype. They are conducting additional rounds of crosses to determine whether resistance is linked to the EVE and to investigate details of maternal vs. paternal transmission. This work was funded under NSF ISO 2048214. No animal or human studies were performed.

Shelby Cagle, Arielle Glass, Corey Campbell, Emma Harris, & Rebekah C. Kading (Colorado State University, Department of Microbiology, Immunology and Pathology, Center for Vector-Borne Infectious Diseases) reported on their strategy to determine how temperature impacts vertical and transstadial transmission rates of Rift valley fever virus in *Culex tarsalis* and *Aedes aegypti* mosquitoes. Rift valley fever virus (RVFV) is an emerging zoonotic, mosquito-borne virus that can cause encephalitic, neurological and/or hemorrhagic disease in sheep, cattle and humans. RVFV (*Phlebovirus*) is transmissible vertically within mosquito populations, through mosquito bites or by aerosolization of viral particles. RVFV is classified as a Select Agent due to its pathogenicity and because it poses a significant risk to human and animal health, which can be detrimental to society, industry, and the economy. Though endemic to East Africa, RVFV is well-poised for introduction to the United States due to human travel, and vector competence has been demonstrated for many mosquito species native to North America. The factors that govern how efficiently mosquito species transmit RVFV transstadially (parent to offspring) is a gap in our current understanding of interepidemic viral maintenance. In particular, understanding how environmental conditions such as temperature may modulate transovarial transmission of RVFV is not yet understood. Transovarial transmission is one mechanism through which viruses persist in the environment across seasons. To elucidate how larval rearing temperature can affect transovarial transmission of RVFV from Culex tarsalis and Aedes aegypti mosquitoes to their progeny, mosquitoes were given an infectious blood meal of RVFV strain KEN128B-15. Seven days after oviposition, they were subsequently fed an uninfected blood meal, and progeny from this second gonotrophic cycle were reared to examine viral load and infection prevalence at each developmental stage. This experiment will be repeated at 28C, 18C and 32C rearing temperatures. The results of these ongoing experiments were presented, specifically, infection rates and viral loads of Culex tarsalis and Aedes aegypti at 28 °C. The authors hypothesized that larval habitat temperature influences the efficiency of transstadial transmission. Through this study, they hope to gain crucial insight into vector competence that can be applied to other mosquito-borne disease systems. This work was funded by NIH project R21 AI148896. No human or animal studies were performed.

Samantha Courtney reported alongside Bekah McMinn, Emily Fitzmeyer, James Weger-Lucarelli, and Gregory D. Ebel (Dept. of Microbiology, Immunology, and Pathology, Colorado State University) on an effective strategy for analyzing Powassan virus transmission dynamics through RNA barcoding. Powassan virus (POWV) is an emerging tick-borne virus that can cause severe neurologic disease including encephalitis and meningitis. POWV is classified into genetically defined lineages: Powassan virus (POWV, Lineage I) and deer tick virus (DTV, Lineage II). DTV, transmitted by black-legged ticks (*Ixodes scapularis*), poses a significant threat to human health because *I. scapularis* are common in highly trafficked wooded areas of the northeastern and north central US. The evolutionary forces exerted on POWV during transmission and pathogenesis are poorly understood. To assess virus population structure during transmission, Courtney et al. developed barcoded viruses to quantitatively measure population bottlenecks in cell culture, arthropods, and vertebrates. In general, barcoded viruses are engineered to contain a series of synonymous nucleotide substitutions that facilitate efficient and cost-effective measurements of the stochastic reductions in virus population size that occur during virus transmission by arthropods. POWV barcoded virus was created by making changes to 11 consecutive codons in the POWV NS2a coding sequence, resulting in 4^11^ (~4.2 million) possible barcodes. DNA containing the synthesized barcode region was inserted into a DTV infectious clone and virus was successfully rescued and propagated in BHK-21 (ATCC CCL-10, baby hamster kidney) cells to high titers (1 × 10^5^ PFU/mL). To confirm the barcode sequence in their new stock, they sequenced the barcoded region and observed 11 degenerate nucleotides as expected. Currently, the authors are preparing to measure the diversity of the barcoded stock via next-generation sequencing. Further optimization of their library preparation is needed to sequence the virus at high depth. Overall, barcoded viruses are a useful genetic tool, and they plan to use this in tick and mouse studies to assess POWV population dynamics during various transmission modalities. Funding was provided by NIH project number 5R01AI137424-04. All animal studies will be performed following guidelines and protocols approved by the Institutional Animal Care and Use Committee of Colorado State University.

Kaleb Davis, Natalie Wickenkamp, Julius Stuart, Arielle Glass, Christopher Snow, and Rebekah C. Kading (Dept. of Microbiology, Immunology, and Pathology, Colorado State University) reported a novel microcrystal DNA marking technique, which can be applied to the tracking of mosquito dispersal. The flight patterns of mosquitoes are a long-standing area of interest for arbovirus researchers. Currently capture, mark, release, and recapture methods are used to collect these data. However, these methods, which frequently rely on fluorescent dyes and or powders, are limited in the number of markers available and can negatively impact the survivability of the marked mosquitoes. The authors’ novel method to mark mosquitoes as larvae uses a microscopic crystalline protein which can sequester DNA within its structure and protect it in a wide array of environmental conditions before releasing it under laboratory conditions for recovery. These microcrystals are loaded with a unique synthetic DNA barcode prior to deployment which can be tied to important information such as the date of deployment, and geographic region. Larval-stage mosquitoes are capable of ingesting DNA-laced microcrystals which will persist within the mosquito into adulthood where they can be recovered. The integration of these barcodes into existing targeted mosquito control networks will allow the tracing of infected mosquitoes from the capture site to the site of larval heritage allowing more focused targeting of existing larval control efforts. This research was supported by NIH/NCATS Colorado CTSA Grant Number UL1TR002535 and NIH R21 AI146740. Its contents are the authors’ sole responsibility and do not necessarily represent official NIH views. This work was also supported in part by Animal Health and Disease Grant No. 2021-01/Project Accession No. 1027096 from the USDA National Institute of Food and Agriculture. No animal or human studies were performed.

### 2.4. Applied Strategies for Addressing Needs in Virology

Sierra Mikula, Jordan A. Powers, Christin Goodman, Holly R. Hughes, Brad J. Biggerstaff, Brent S. Davis, Amanda E. Calvert from the U.S. Centers for Disease Control and Prevention described the Development of an anti-Cache Valley virus human-murine chimeric IgM for use as a positive control in serodiagnostics. Cache Valley virus (CVV) is a mosquito-borne virus in the genus *Orthobunyavirus*, family *Peribunyaviridae* that has recently emerged as a viral pathogen causing severe disease in a handful of patients. While CVV is the causative agent of fetal death and severe malformations in livestock, its teratogenicity in humans remains largely undetermined. Mikula et al. recently developed hybridomas secreting anti-CVV murine monoclonal antibodies (Mabs) for the development of an IgM antibody capture enzyme-linked immunosorbent assay (MAC-ELISA) for use in human serodiagnosis. However, a lack of bulk human positive-control sera hinders testing in routine diagnostic algorithms. Here, the authors describe the development of a HEK-293 cell line that constitutively expresses a human-murine chimeric antibody with the variable regions of Mab CVV17 and the constant region of the human IgM. The HEK293 cell line (CVV17-hIgM) secreting the engineered human IgM produced high concentrations of antibody with high reactivity to CVV (endpoint ≥ 0.03 ng/mL) and demonstrated similar reactivity compared with the standard human immune sera in the assay. The CVV17-hIgM cell line also showed stable antibody production throughout 10 cell passages. The CVV MAC-ELISA with the engineered positive control described here will help expand diagnostic capacity and facilitate research to increase understanding of Cache Valley virus disease prevalence and the virus’ impact on human populations. The findings and conclusions in this report are those of the authors and do not necessarily represent the official position of the CDC. Ethics approval for use of previously collected human diagnostic specimens was obtained from CDC’s Human Subjects Institutional Review Board (CDC IRB# 6773). The positive control specimen was delinked from any personal identifiers prior to the commencement of this study. This project was supported, in part, by an appointment to the Research Participation Program at the Centers for Disease Control and Prevention administered by the Oak Ridge Institute for Science and Education through an interagency agreement between the U.S. Department of Energy and the Centers for Disease Control and Prevention.

Scott Seitz ^1,2,4^, described his work in collaboration with Lauren E. Brown ^2^, Paul Marcyk ^2^, Andrew de Los Santos ^2^, Claire Marie Filone ^1^, Scott E. Schaus ^2^, Ga Lee ^4^, Randall J. Cohrs ^4^, John H. Connor ^1,2^ (Dept. of Microbiology, Boston University ^1^. National Emerging Infectious Disease Laboratories, Boston University ^2^. Dept. Chemistry, Boston University ^3^. Dept. of Neurology, University of Colorado Anschutz ^4^) his work in two separate drug studies, which took place initially in Aurora and then moved to Boston. Herpesviruses are a ubiquitous pathogen known to cause several types of disease. The international space station (ISS) is a unique structure providing various stressors that differ from our terrestrial existence. A study was started to look at the effects of these extraterrestrial inputs on overall drug stability. Two types of drugs were chosen due to the commonality of outbreaks on ISS: antibiotics, and acyclovir, a nucleoside analog used against both herpes simplex 1 and varicella zoster virus, two of the most common maladies on ISS. The study concluded that although some efficacy is lost compared to an Earth-stored matching batch, the reduction was not substantial enough for requiring more frequent drug resupply. It was this first experience in drug testing that led to an interest in developing drugs against diseases in the field of public health. (Boston MA, circa 2021) Orthopoxviruses are responsible for some of the most devastating diseases known to humankind including both smallpox and monkeypox. Seitz et al. have been working on developing small molecule inhibitors of poxvirus and have used non-nucleosidal pyridopyrimidinone (PDPMs) as a scaffold that led to our discovery of 6128; a molecule with broad activity against poxviruses, including variola. As the drugs are developed in collaboration with BU’s chemistry department, they have been able to characterize how chemical modifications affect anti-viral activity. These chemical modifications led to two compounds that show a 10–100 fold increase in efficacy over 6128, with activities in the nanomolar. No animal or human studies were completed in this study.

Clara Reasoner, Bradly Burke, Wenjun Ma and Tony Schountz (Dept. of Microbiology, Immunology, and Pathology, Colorado State University) described the generation of competent Jamaican Fruit Bat (*Artibeus jamaicensis*) Dendritic Cells and Macrophages that could improve analysis of bat immune responses. New World fruit bats serve as reservoirs for rabies virus, H17N10 and H18N11 bat influenza A viruses, and coronaviruses. Because bat influenza A viruses were first detected in New World fruit bats in Guatemala and subsequently Peru and Brazil, these species likely serve as the natural reservoir hosts. Unlike other Influenza A viruses that use sialic acid residues for cellular binding, H18N11 uses MHC class II beta chains for cellular entry. Due to the high sequence conservation of MHC molecules, the use of MHC class II molecules as an entry mediator leads to the possibility of broad vertebrate tropism. Therefore, the characterization and study of H18N11 infection in professional antigen-presenting cells (APC) from New World fruit bats will lead to a greater understanding of the mechanisms by which these species control viral infections and how H18N11 infects MHC class II positive cells. APCs were generated with Jamaican fruit bat (*Artibeus jamaicensis)* bone marrow-derived hematopoietic stem cells (HSC) cultured in Jamaican fruit bat GM-CSF or Egyptian rousette fruit bat (*Rousettus aegyptiacus*) M-CSF. Cells were characterized using flow cytometry and challenged with H18N11. Cultured dendritic cells were found to express MHC-II, which signifies their role as APCs. Despite their expression of MHC class II, the cells were not permissive to infection with H18N11, suggesting that another molecule may be required for successful H18N11 entry. Through culture of Jamaican fruit bat HSCs with GM-CSF and M-CSF, functional dendritic cells and macrophages were generated. These cells are important for the activation and augmentation of the adaptive immune response to viruses. The cells cultured can be used to define function and test viral susceptibility and permissibility. Cultured dendritic cells can also be used to stimulate T cells in culture with antigens of Interest, in order to gauge the bat adaptive immune response to viruses of interest. It is important to understand the cellular characteristics of antigen-presenting cells (APCs) in New World fruit bats, particularly in relation to bat influenza, which uses MHC II as an entry mediator. This project was supported by the National Institute of Allergy and Infectious diseases (R01AI134768). All animal studies were performed following guidelines and protocols approved by the Institutional Animal Care and Use Committee of Colorado State University.

Erica Suchman (Department of Microbiology, Immunology and Pathology, Colorado State University) described her methods for teaching of medical and molecular virology, with an emphasis on effective strategies that leave lasting impressions on students, beyond mere memorization for exams. Her pitch was as follows: “Are you tired of watching students play on their phones while you give an amazing lecture? This talk will describe my journey to “flipping” my large (over 60 students) virology course during the pandemic. I will describe what that looks like, what it took to do this, and mechanisms for increasing student engagement and learning in large lecture virology courses will be demonstrated. In class, students analyze trends in taxonomy for RNA and DNA viruses, prepare concept maps (viral enzymes, immune responses, complete works diagnostic tests, viral protein production and replication strategies) and group exams to move past memorization. Examples of materials used were shared with the attendees.

### 2.5. Advances in Prion Disease Biology and Pathogenesis

Joseph DeFranco ^1^, Sehun Kim ^1^, Jifeng Bian ^1,2^ Jenna Crowell ^1^, and Glenn Telling ^1^ (^1^ Prion Research Center, the Department of Microbiology, Immunology, and Pathology, Colorado State University, Fort Collins, Colorado. ^2^ Virus and Prion Research Unit, USDA National Animal Disease Center, Ames, Iowa) presented their work on Diverse prion strains resulting from selective propagation of distinct quasispecies conformations in different host compartments. Chronic wasting disease (CWD) is a rapidly spreading, uncontrolled prion disease in wild and captive cervids in North America, Europe, and Asia. This pathogen transmits through bodily fluids, shedding skin during infection, is invariably fatal, and has incomparable, robust environmental persistence. Although it is assumed that the primary mode of natural CWD is likely horizontal transmission, either through direct contraction between cervids or indirect infection via contaminated fomites in the environment, there is still limited understanding of how these different transmission routes affect disease pathogenesis. Despite PrP^Sc^ (i.e., the pathogenic form of host-encoded PrP^C^) lacking informational nucleic acids, prions share strain diversity analogous to conventional pathogens. These strain properties affect prion infectivity and pathogenesis, PrP^Sc^ biochemical properties, and host-range dynamics. It is well established that different routes of prion exposure engender varying disease incubation times; however, this was attributed to the duration of time needed for the pathogen to enter the central nervous system to cause disease. Using newly developed gene-targeted models, the research group has found that the route of inoculation has a profound effect on prion pathogenesis. Specifically, they have discovered that, at the terminal stage of disease, there are distinct biochemical conformations of the protein, varied titers of the infectious pathogen, and different depositions of prions in the brain. These data suggest that unique prion strains are propagated as a result of the route of prion exposure. This research is critical to understanding the natural transmission and zoonotic potential of CWD, as well as fostering the study of prion biology. The study was funded by The National Institutes of Health grant numbers R01NS121682 and PO1-0011877A and the Anschutz Foundation (AF) under grant number 6476430. All animal studies were performed following guidelines and protocols approved by the Institutional Animal Care and Use Committee of Colorado State University. No human studies were performed.

Glenn Telling ^1^ together with Juliana Sun ^1^, Sehun Kim ^1^, Jenna Crowell ^1^, Bailey K. Webster ^1^, Emma K. Raisley ^1^, Diana C. Lowe ^1^, Jifeng Bian ^1^, Hyun Joo Sohn ^2^, Hae-Eun Kang ^2^, Sirkka-Liisa Korpenfelt ^3^, Maria Nöremark ^4^, and Sylvie Benestad ^5^ (^1^ Dept. of Microbiology, Immunology, and Pathology and Prion Research Center (PRC), Colorado State University, USA; ^2^ Animal and Plant Quarantine Agency (QIA), 177 Hyeoksin 8-ro, Gimcheon-si, Gyeongsangbukdo 39660, Republic of Korea; ^3^ Finnish Food Safety Authority Evira, Research and Laboratory Services Department, Virology Research Unit, Mustialankatu 3, Fi-00790 Helsinki, Finland; ^4^ Department of Disease Control and Epidemiology, SVA, National Veterinay Institute, Uppsala, Sweden; ^5^ Norwegian Veterinary Institute, Postboks 750 Sentrum, 0160 Oslo, Norway) discussed their studies on how emergent Nordic CWD prion strains are distinct from and more diverse than North American and South Korean CWD. The prion hypothesis embodies the radical concept that prion proteins (PrP) contain information necessary and sufficient for infectious replication within their structure, thus obviating the requirement for genomic material. Accordingly, primary structural differences between PrP from different species have pronounced effects on disease outcomes. Prions causing chronic wasting disease (CWD) have an unusually wide host-range, and cervid PrP coding sequences (CerPrP) are generally invariant except at codon 226 [deer encode glutamine (Q), while North American elk encode glutamate (E)]. To assess the effects of this primary structural difference on CWD outcomes we created gene targeted (Gt) mice in which the murine PrP coding sequence was replaced with CerPrP-Q226 or CerPrP-E226, referred to as GtQ and GtE mice. They brought to discussion how their previous work in Gt mice showed that residue 226 controls the selection of distinct North American CWD prion strains. Here, they aimed to assess the responses of GtE and GtQ mice to emergent CWD prions from Nordic countries and South Korea, and to compare their properties with North American CWD. The team has intracerebrally or intraperitoneally inoculated mice with homogenates of frozen brain and lymphoid tissue materials from Norwegian, Swedish, Finnish, and South Korean CWD cases. Then, they assessed conventional measures of prion strain properties including differences in susceptibilities and disease kinetics, neuropathology, lymphotropism, and various biochemical and cell biological assessments of the resultant prions. Further, properties of these prions after iterative transmissions in mice were also assessed. They reported that while prions causing CWD in South Korea share properties with epidemic CWD in North America, newly emergent forms of CWD in Nordic countries are distinct from North American CWD and show high levels of strain diversity and instability. The surprisingly diverse spectrum of unstable Nordic CWD strains stands in contrast the relatively consistent and stable strain profile of established CWD in North America. All animal studies were performed following guidelines and protocols approved by the Institutional Animal Care and Use Committee of Colorado State University. No human studies were performed.

Alyssa J. Block ^1^, Ronald A. Shikiya ^1^, Thomas E. Eckland ^1^, Anthony E. Kincaid ^2^, Romilly Benedict ^3^, Ryan W. Walters ^4^, Jiyan Ma ^5^, and Jason C. Bartz ^1^ (^1^ Creighton University, Department of Medical Microbiology and Immunology, United States; ^2^ Creighton University, Department of Pharmacy Science, United States; ^3^ Michigan State University, Department of Plant, Soil, and Microbial Sciences, United States; ^4^ Creighton University, Department of Medicine, United States; ^5^ Van Andel Research Institute, Center for Neurodegenerative Science, United States) reported their studies on Mechanisms of adaptation of synthetic prions in hamsters. Prion diseases are a group of neurodegenerative disorders that affect mammals, including humans. Prions are comprised of PrP^Sc^, the self-templating pathogenic conformation of the cellular prion protein, PrP^C^. Synthetic prions, generated in vitro from minimal components, cause bona fide prion disease in animals expressing syngeneic PrP^C^, albeit with extended, variable incubation periods and incomplete attack rates. In contrast, murine synthetic prions (MSP) formed via PMCA with minimal cofactors readily infected mice and hamsters. It is unknown whether hamster synthetic prions (HSP) formed under the same conditions are also highly infectious for hamsters. To investigate this, hamster WT or mutant (Δ54, Δ54/139, 139/205) synthetic prions were inoculated into Syrian hamsters. All hamsters inoculated with HSP did not develop clinical signs of prion disease. Western blot analysis of brain homogenate from HSP^WT^- and HSP^Δ54^-infected hamsters identified protease-resistant PrP^Sc^, suggesting subclinical infection. Serial intraspecies transmission resulted in clinical disease at the second passage followed by slow adaptation to hamsters, including changes in conformational stability during serial passage. Transmission and slow adaptation of the HSP to hamsters stands in stark contrast to the MSP, which efficiently crossed the species barrier and rapidly adapted to hamsters. These data suggest the HSP, in contrast to the MSP, are not comprised of *bona fide* PrP^Sc^ and instead generate authentic PrP^Sc^ through the process of deformed templating. Combined, differences in infectivity between the HSP and MSP in hamsters suggest that under identical formation conditions, the amino acid sequence of PrP dictates the generation of authentic PrP^Sc^. All animal studies were performed following guidelines and protocols approved by the Institutional Animal Care and Use Committee of Creighton University. The work was funded through the National Institutes of Health National Institute of Neurological Disorders and Stroke (R01NS103763) and the National Institute of Allergy and Infectious Disease (2P01 AI077774).

Joseph Hrdlicka together with Qi Yuan, and Jason Bartz from Creighton University, Department of Medical Microbiology and Immunology, United States discussed their efforts on Evaluation of real-time quaking-induced conversion for prion strain discrimination and infectivity

Prions are amyloid-forming proteins capable of causing neurodegenerative disease through the conversion of the normal prion protein (PrPC) to a misfolded conformation (PrPSc). Chronic wasting disease (CWD) is a prion disease that affects cervids, is inevitably fatal, highly contagious, and has an unknown host range, possibly zoonotic. Therefore, the detection of CWD prions in early stages is critical for controlling disease transmission and public health. Real-time quaking-induced conversion (RT-QuIC) has been proven as a reliable assay in detecting amyloid fibril formation and diagnosing prion diseases. Currently, there is limited information about the use of RT-QuIC in quantitatively determining prion infectivity and strain discrimination. With prion diseases such as CWD increasing in prevalence, measurements requiring less time and resources with potential to assess infectivity and strain discrimination are crucial. They hypothesized RT-QuIC can quantify prion infectivity and discriminate prion strains. To investigate the ability of RT-QuIC in assessing prion infectivity, they compared the lethal dose (LD50) to the seeding dose (SD50) value, or the dose of prion generating a positive signal in RT-QuIC for 50% of tested samples, using two distinct hamster prion strains known as hyper transmissible mink encephalopathy (HY TME) and drowsy transmissible mink encephalopathy (DY TME). To investigate the ability of RT-QuIC to discriminate strains, the SD50 difference and LD50 difference between the two hamster prion strains were compared. The team reported the SD50 values of HY TME from RT-QuIC results to be strongly comparable to the LD50 values from animal bioassay. RT-QuIC did not accurately assess prion infectivity for DY TME. Comparison of SD50 values from RT-QuIC to LD50 values from animal bioassay indicated the ability of RT-QuIC to discriminate between the two distinct prion strains. Based on their results, RT-QuIC can be a valuable tool in further developing quantitative analysis of prions and become a vital tool for the management and surveillance of CWD. No animal or human studies were performed.

Koshy S ^1^, Shikiya R ^1^, Kincaid A ^2^ and Bartz J ^1^ from the Department of Medical Microbiology and Immunology, Creighton University ^1^ and Department of Pharmacy Science, Creighton University ^2^ elaborated their findings on axonal transport of PrP^Sc^. Prion diseases are invariably fatal neurodegenerative disorders caused by the misfolded, infectious prion protein (PrP^Sc^). When peripherally acquired, PrP^Sc^ is thought to spread via slow axonal transport based on immunohistochemistry, immunoblot analysis of tissue homogenates, detection of spongiosis, or the presence of prion infectivity using animal bioassay. A shortcoming of these methods is that they have low sensitivity and measure both inoculum PrP^Sc^ and newly replicated PrP^Sc^, confounding the observed PrP^Sc^ transport rates. In this study, the researchers utilized highly sensitive PMCA to measure PrP^Sc^ transport in a prion replication-deficient system to accurately calculate PrP^Sc^ transport rate and investigate the role of normal endogenous prion protein (PrP^C^) in PrP^Sc^ axonal transport. PMCA was able to detect down to 10^−12^ μg eq. of hamster Hyper (HY) PrP^Sc^ in hamster substrate while the conversion efficiency in mouse substrate was substantially lower. Additionally, negative control unseeded reactions and reactions seeded with uninfected mouse tissue failed to amplify PrP^Sc^ in hamster substrate. After two PMCA rounds in hamster substrate, the group failed to detect hamster 263K PrP^Sc^ in uninoculated mouse sciatic nerve (ScN) 24 h (post-infection) p.i. but PrP^Sc^ was detected in the inoculated mouse ScN and lumbar spinal cord (SC) 24 h p.i. Based on a distance from the inoculation point to the lumbar SC, PrP^Sc^ transport rate was calculated as at least 25 mm/day, well above established slow transport rates (0.3–8 mm/day) and within the accepted rates of fast axonal transport. They concluded that these data suggest a potential PrP^Sc^ fast axonal transport mechanism. All animal studies were performed following guidelines and protocols approved by the Institutional Animal Care and Use Committee of Creighton University and the study was funded by NIH NINDS 1R01NS107246.

Erin McNulty, together with Amy Nalls, Candace Mathiason from the Department of Microbiology, Immunology, and Pathology, Colorado State University presented their studies on the multigenerational transmission of CWD prions from mother to offspring using the Reeve’s muntjac deer. Chronic wasting disease (CWD) continues to demonstrate geographic expansion, now found in captive and/or free-range cervid populations in North America, South Korea, and Scandinavia. While horizontal transmission is credited for much of the spread of CWD, few studies have monitored the transmission of this disease from mother to offspring, but it is gaining momentum as a valid hypothesis for facile transmission. CWD-infected muntjac dams are able to become pregnant, carry, deliver and rear offspring during the long asymptomatic phase of prion infection. The group has demonstrated that CWD prions can be transmitted from mother to first-generation offspring leading to prion infection and subsequent development of TSE disease and that transmission occurs during gestation. They have also demonstrated that there are infection prions within the pregnancy microenvironment by bioassay (uterus, birthing fluids, and placentomes). They assessed tissues harvested from clinical terminal muntjac dams, in-uterine collections of late-term fetuses, and full-term second-generation nonviable muntjac offspring for infectivity by mouse bioassay. Transgenic mice expressing the cervid prion protein, Tg(CerPrP-E226)5037+/− were either IC inoculated with 10% ovary or mammary gland/LN homogenate, or IP-inoculated with PMCA-amplified RAMALT, ileum, or obex from first generation fetuses and lung, mammary gland, kidney or uterus from nonviable 2nd generation muntjac offspring.

Their results showed all muntjac dam tissue inoculated mice, all first-generation PMCA-amplified tissue inoculated mice and all mice inoculated with PMCA-amplified tissue from second-generation offspring developed signs consistent with TSE disease, and were confirmed CWD positive by Western blot and RT-QuIC. Negative control mice remained healthy and TSE-free for the same duration.

Finally, their data indicate that: (1) multigenerational transmission of infectious CWD prions from mother-to-offspring may be possible and (2) early and persistent exposure of the developing embryo to infectious CWD prions in the uterine microenvironment may help explain the facile transmission of CWD in the native host. All animal studies were performed following guidelines and protocols approved by the Institutional Animal Care and Use Committee of Colorado State University. Funding was provided via NIH R01AI093634

Samantha Scherner, Amy Nalls, Erin McNulty, Nathanial Denkers and Candace Mathiason from the Department of Microbiology, Immunology and Pathology, Colorado State University discussed their findings on determining specificity, sensitivity and consistency of the RT-QuIC assay. Real-time-quaking induced conversion (RT-QuIC) is contemporary in vitro amplification assay used to detect low concentrations of misfolded proteins associated with amyloid-generating or prion diseases. RT-QuIC sensitivity can be 106-fold more sensitive than conventional prion detection assays, including ELISA and immunohistochemistry (IHC). Prion seeds (PrP^RES^) present in tissues and fluids of prion-infected hosts are amplified by RT-QuIC when the misfolded seed coerces a recombinant syrian hamster prion protein (rhaPrP) into the misfolded shape. Real-time readout is achieved by the intercalation of a fluorescent dye, Thioflavin T, between the growing amyloid fibrils. To determine the consistency, specificity and stability of recombinant protein batches used in RT-QuIC they assessed rhaPrP fidelity for twelve batches of in-house generated protein over a two-month period. At two-week intervals, they assessed RTQuIC quality control at 42C and 55C using positive and negative chronic wasting disease (CWD) brain homogenate dilutional series and SDS-only controls. Data collected was compiled into stacked graphs for all twelve rhaPrP batches. Their findings indicate that after two months rhaPrP begins to spontaneously produce amyloid fibrils in negative controls. Using these data, they to established a shelf life of six-seven weeks/rhaPrP batch to maintain the specificity of the RT-QuIC assay. Their study has provided crucial information resulting in a high level of confidence for consistency between rhaPrP batches to maintain specificity without spontaneous amyloid generation, thus supporting the use of this ultra-sensitive in vitro amplification assay as a biomarker to reveal the presence and mechanisms associated prion and prion-like disorders. All animal studies were performed following guidelines and protocols approved by the Institutional Animal Care and Use Committee of Colorado State University and the work was funded by NIH RO1-NS061902-09R, PO1-AI077774, RO1-AI112956-06 grants.

M.L. Tyer ^1^ together with Xutong Shi ^1^, Juliana Sun ^1^, Sehun Kim ^1^, Sylvie Benestad ^2^ and Glenn Telling ^1^ (^1^ Department of Microbiology, Immunology and Pathology Colorado State University. Fort Collins, Colorado, ^2^ Norwegian Veterinary Institute. Oslo, Norway) discussed their work on the exploratory use of RT-QuIC to quantify and compare kinetics of amyloid formation in North American and Scandinavian CWD prions. Chronic wasting disease (CWD) is a transmissible, universally fatal neurodegenerative disease caused by the pathogenic misfolding of prion protein. CWD is endemic to North America (NA), and most recently has been identified in Scandinavian cervids. Interestingly, isolates collected from Scandinavian CWD cases have been shown to possess unique strain characteristics. Previous research from their group has shown Scandinavian CWD is etiologically distinct from its North American counterpart, which may account for these strain differences.

Here, the researchers seek to further characterize the kinetic properties of Norwegian CWD (NorCWD) isolates through real-time quaking-induced conversion (RT-QuIC) assay, a method for quantifying the rate at which a prion seed can form amyloid from a standard substrate. Their preliminary results show that Nor red deer and moose isolates display variable amyloid formation rates within technical replicates, with little discernable linear range over a dilution series. This is distinct from NA elk and mule deer, which display a predictable linear range over 10^−4^ to 10^−7^ titrations. Notably, the NorCWD isolates converted substrate at a faster rate than seeds taken from NA. Taken together, these results indicate that the distinct properties of NorCWD can be recapitulated using a standard in vitro assay with recombinant Syrian hamster PrP as a substrate. The presenters conclude that these preliminary data will guide the direction of their future research investigating features influencing NoCWD pathogenicity. All animal studies were performed following guidelines and protocols approved by the Institutional Animal Care and Use Committee of Colorado State University.

Qi Yuan ^1^, Gage Rowden ^2^, Tiffany M. Wolf ^3^, Marc D. Schwabenlander ^2^, Peter A. Larsen ^2^, Shannon L. Bartelt-Hunt ^4^ and Jason C. Bartz ^1^ (^1^ Department of Medical Microbiology and Immunology, Creighton University, Omaha, NE 68178, USA, ^2^ Department of Veterinary and Biomedical Sciences, University of Minnesota, Saint Paul, MN 55108, USA, ^3^ Department of Veterinary Population Medicine, University of Minnesota, Saint Paul, MN 55108, USA, ^4^ Department of Civil and Environmental Engineering, Peter Kiewit Institute, University of Nebraska-Lincoln, Omaha, NE 68182, USA) elaborated their work on the sensitive detection of chronic wasting disease prions recovered from environmentally relevant surfaces. Chronic wasting disease (CWD) has been identified in 30 states in the United States, four provinces in Canada, and recently emerged in Scandinavia. The association of CWD prions with environmental materials such as soil, plants, and surfaces may enhance the persistence of CWD prion infectivity in the environment exacerbating disease transmission. Identifying and quantifying CWD prions in the environment is significant for prion monitoring and disease transmission control. A systematic method for CWD prion quantification from associated environmental materials, however, does not exist. In this study, they developed an innovative method for extracting prions from swabs and recovering CWD prions swabbed from different types of surfaces including glass, stainless steel, and wood. We found that samples dried on swabs were unfavorable for prion extraction, with the greatest prion recovery from wet swabs. Using this swabbing technique, the recovery of CWD prions dried to glass or stainless steel was approximately 30% in most cases, whereas that from wood was undetectable by conventional prion immunodetection techniques. Real-time quake-induced conversion (RT-QuIC) analysis of these same samples resulted in an increase in the detection limit of CWD prions from stainless steel by 4 orders of magnitude. More importantly, the RT-QuIC detection of CWD prions recovered from stainless steel surfaces using this method was similar to the original CWD prion load applied to the surface. This combined surface swabbing and RT-QuIC detection method provides an ultrasensitive means for prion detection across many settings and applications. No animal or human studies were performed. This study was supported by the National Institutes of Health (grant number P012P01AI077774 to J.C.B.) and the National Science Foundation (award number CBET-1149424 to S.L.B.)

Jesse Cole ^1^ along with Erin McNulty ^1^, Audrey Sandoval ^1^, Amy Nalls ^1^, Michael Chamberlain ^2^, Mark Ruder ^3^, Joseph Westrich ^1^ and Candace Mathiason ^1^ (^1^ Dept. of Microbiology, Immunology, and Pathology, Colorado State University, ^2^ Warnell School of Forestry, University of Georgia, Athens, ^3^ Southeastern Cooperative Wildlife Disease Study, University of Georgia, Athens) discussed their work on Transcriptional and Translational Variation of Prion Protein (PRNP) expression in White-tailed Deer Fetuses. Chronic Wasting Disease (CWD) is a rapidly spreading, invariably fatal protein misfolding or prion disease of cervid species (deer, elk, moose and reindeer), that has expanded in strain designation, geographic location, and host range. CWD is the most efficiently transmitted of all the prion diseases and is currently detected in captive and free-ranging cervid populations in 30 U.S. States, 4 Canadian Provinces, Europe, and Asia. Transmission of CWD has been largely attributed to horizontal transmission by direct animal-to-animal contact with bodily secretions (saliva, blood, urine and feces), and by indirect contact with the infectious agent shed in these products to the environment. A relatively understudied route of CWD transmission is that from mother to offspring during pregnancy, or vertical transmission. The research group has previously demonstrated that CWD vertical transmission occurs in muntjac (experimental) and in free-ranging elk and white-tailed deer populations. The infectious agent has been demonstrated within the pregnancy microenvironment (ovary, uterus, placentome, amniotic fluid), as well as within fetal tissues. Prion diseases manifest as a posttranslational modification of the host-encoded cellular prion protein (PrP^c^) as it converts to an aberrantly folded, partially proteinase-resistant disease-associated form (PrP^Sc^). To begin to unravel mechanisms associated with CWD vertical transmission, Jesse and the group investigated Prnp expression (transcripts and protein) in pregnancy and fetal tissues to determine the relative abundance of prion-converting substrate present at the maternal-fetal interface. Converting substrate is undoubtedly required to initiate CWD vertical transmission. In the study, tissues (fetal spleen, brain, liver and pregnancy umbilicus, placentome) harvested from 9 in utero-derived free-ranging white-tailed deer pregnancies with known dam CWD status were examined by quantitative reverse transcription PCR (RT-qPCR) and Western blot to assess Prnp and PrP^c^ expression. The authors mentioned that these studies will provide the basis for continued studies to determine how CWD prions traffic across the placental barrier to infect the developing fetus. The study was funded by NIH- 5T32GM136628-03. All animal studies were performed following guidelines and protocols approved by the Institutional Animal Care and Use Committee of Colorado State University

## Data Availability

No new data were created or analyzed in this study. Data sharing is not applicable to this article.

